# Genome-wide analysis of non-coding RNA reveals the role of a novel miR319c for tuber dormancy release process in potato

**DOI:** 10.1093/hr/uhae303

**Published:** 2024-10-30

**Authors:** Shengyan Liu, Jiangwei Yang, Ning Zhang, Huaijun Si

**Affiliations:** State Key Laboratory of Aridland Crop Science, Gansu Agricultural University, Yingmencun No.1, Anning District, Lanzhou 730070, China; College of Agronomy, Gansu Agricultural University, Yingmencun No.1, Anning District, Lanzhou 730070, China; State Key Laboratory of Aridland Crop Science, Gansu Agricultural University, Yingmencun No.1, Anning District, Lanzhou 730070, China; College of Life Science and Technology, Gansu Agricultural University, Yingmencun No.1, Anning District, Lanzhou 730070, China; State Key Laboratory of Aridland Crop Science, Gansu Agricultural University, Yingmencun No.1, Anning District, Lanzhou 730070, China; College of Life Science and Technology, Gansu Agricultural University, Yingmencun No.1, Anning District, Lanzhou 730070, China; State Key Laboratory of Aridland Crop Science, Gansu Agricultural University, Yingmencun No.1, Anning District, Lanzhou 730070, China; College of Life Science and Technology, Gansu Agricultural University, Yingmencun No.1, Anning District, Lanzhou 730070, China

## Abstract

Tuber dormancy and sprouting are significant for potato cultivation, storage, and processing. Although the substantial role of microRNAs (miRNAs) in some biological processes has been recognized, the critical role of miRNA in breaking potato tuber dormancy is not well understood to date. In this investigation, we expand research on miRNA-mediated gene regulation in tuber dormancy release. In this work, 204 known and 192 novel miRNAs were identified. One hundred thirty-six differentially expressed miRNAs (DE-miRNAs) were also screened out, of which 56 DE-miRNAs were regulated by temperature during tuber dormancy release. Additionally, degradome sequencing revealed that 821 target genes for 202 miRNAs were discovered. Among them, 63 target genes and 48 miRNAs were predicted to be involved in plant hormone signaling pathways. This study used degradome sequencing, tobacco cotransformation system, and β-glucuronidase (GUS) staining technology to confirm that stu-miR319c can target *StTCP26* and *StTCP27* and effectively suppress their expression. The transgenic approach exhibited that stu-miR319c overexpressed tubers sprouted in advance, while silent expression of stu-miR319c showed delayed sprouting. Treatment of wild-type tubers with exogenous MeJA revealed that 1 mg/L MeJA significantly broke dormancy and enhanced potato sprouting ability. Furthermore, transgenic tubers revealed variance in jasmonic acid (JA) content and relative expression of genes associated with the JA synthesis pathway, including *StAOC*, *StLOX2*, and *StLOX4*, suggesting that the miR319c may participate in the JA pathway to regulate tuber dormancy release. In summary, our research offers evidence that miRNA regulates potato dormancy release and supports the idea that stu-miR319c is a unique epigenetic regulator for dormancy-sprouting transition in potatoes.

## Introduction

Plant dormancy is a condition in which the growth and metabolism of a plant body or its organs are exceedingly slow or temporarily interrupted during a given development phase [1]. There exist multiple manifestations of dormancy. The first is seed dormancy, in which annual or biennial plants primarily use seeds as dormancy organs [2]. The second is bud dormancy, for example, perennial deciduous trees use dormant buds to overwinter [2]. Perennial trees in temperate locations have exceptional growth characteristics of cyclic transition between growth and dormancy with seasonal changes, which are principally carefully governed by photoperiod and temperature [3]. Before the onset of winter, the terminal buds of trees undergo a succession of acclimatization responses, including growth seizure, bud retention, and dormancy. After the terminal bud dormancy, the apical meristem is enveloped by the bud scale structure to endure the cold winter. The dormancy break is facilitated by the persistent low temperatures of winter, which allow the terminal buds to break green and resume growth after sensing the long sunlight conditions [4]. Bud dormancy in temperate plants is a natural and required cyclical process that serves as part of the plant’s defense against cold winter temperatures [2]. The third is the dormancy of roots and underground storage organs, such as potatoes [2]. Potato (*Solanum tuberosum* L.) is the largest noncereal food crop in the world and is crucial to both the global economy and food security [5]. Potato tubers remain dormant for 1–15 weeks after harvest. Potato dormancy is defined as the tuber can’t sprout even in the appropriate temperature, humidity, and dark environment after harvest, and it must take a specific period to sprout, which is known as the dormancy period [6]. Tissue cells at the bud eye in the dormant tuber do not divide. When the dormancy is released, the apical bud meristem of the tuber awakens and sprouts, leading to rapid growth of the buds. Therefore, sprouting also signifies the end of the potato dormancy period [6, 7]. A tuber is deemed germinated when it possesses buds of a minimum of 2 mm in length. The interval between tuber sprouting to seedling emergence is referred to as the sprouting phase [8]. The dormant period, which is a prominent feature of potatoes, greatly influences potato planting and breeding in the early stages and storage in the later stages, while sprouting seriously reduces the commercial value of tubers. The dormancy characteristic of potato tubers plays a vital role in both agricultural and industrial production [9]. Consequently, exploring the key genes [microRNAs (miRNAs)] and hormonal signaling in potato dormancy release and cultivating potato varieties with a suitable dormancy period is of great significance to potato breeding.

The dormancy and sprouting of potato tubers is a multifaceted cellular, hormonal, and physiological process that is affected by various elements, such as environmental conditions, plant hormones, and signaling molecules [10]. Environmental considerations include temperature, humidity, light, external stimuli (germination inhibitors and stimulating chemicals), and ventilation conditions [2]. Postharvest low-temperature storage is a crucial method to extend the annual supply of potatoes. The optimal storage temperature for preventing sprouting growth is between 3 and 4°C. Usually under the temperature of 4–8°C, it can effectively reduce the loss caused by tuber shrinkage, decay, and germination. The determined temperature for stimulating potato sprouting was 16–30°C [11]. It has been reported that low temperatures can enhance the stability of sugar in tubers. Long-term low-temperature storage will lead to sweetening induced by low temperature, while the absence of sugar (energy) is essential to stimulate potato sprouting, which delays the sprouting process [12]. Previous research has demonstrated that various plant hormones are crucial for regulating the dormancy release of potato tubers, such as abscisic acid (ABA), which is the principal component to promote potato tuber dormancy and inhibits sprouting by inhibiting cell division and reduces energy metabolism in the tuber [13]. In contrast to ABA, gibberellins (GAs) promote the sprouting of potato tubers [14]. Furthermore, phytohormones such as cytokinin (CTK), auxin (IAA), and jasmonic acid (JA) also significantly regulate potato postharvest tuber dormancy [15]. Likewise, physiological indices are also very critical indicators for potato tuber dormancy release. In recent years, studies on the metabolism of reactive oxygen species (ROS) and antioxidant enzymes in tuber dormancy have gradually increased. Exogenous hydrogen peroxide (H_2_O_2_) and catalase (CAT) inhibitors both contribute to the early break of dormancy in tubers [16]. With the release of dormancy, the activity of polyphenol oxidase (PPO) gradually decreases, suggesting that PPO is the main respiratory enzyme system during dormancy [17]. Peroxidase (POD) can oxidize IAA side chains, affecting IAA concentration, while IAA can stimulate tuber dormancy breaking [18]. Advanced sequencing technologies have identified several genes involved in potato dormancy regulation. A previous study found that 9797 differentially expressed genes were identified from potato tuber dormancy to sprouting by RNA-seq sequencing, with significant changes in hormone-related genes like IAA, GA, CTK, ethylene (ET), JA, and salicylic acid (SA), highlighting the role of hormones in dormancy regulation [19]. Similarly, heat-stable conditions induced heat sprouts, shortened dormancy after harvesting, and identified genes enriched in the gibberellin biosynthesis pathway [20]. A study uses quantitative phosphorylation proteomics to analyze protein phosphorylation changes during brassinosteroids (BR)-induced potato tuber sprouts, revealing that BR regulate the sprouting mechanism [21]. Previous research has shown that hormonal functions related to potato tuber dormancy and sprouting, such as overexpressing GA20-oxidase, can cause early sprouts in transgenic potato tubers [22]. During dormancy, expression of *NCED1/2* decreases and *CYP707A1* increases [23], while transcripts encoding *LOX* are reduced in dormant tubers [19]. Overexpression of *StSN2* and *StBIN2* negatively regulates BR signaling and maintains tuber dormancy [24].

miRNA, a 20–24 nt non-coding small RNA, is ubiquitous in organisms and regulates plant growth and development by inhibiting target gene activity at post-transcription stages [25, 26]. The miRNAs play a significant role in plant growth and differentiation by regulating different transcription factors (TFs) and hormonal activities. Previous research has shown that to regulate seed dormancy and germination, miR160 down-regulates the *ARF10*, *ARF16*, and *ARF17* target genes and reduces seed sensitivity to ABA [27]. Overexpression of miR159 in *Arabidopsis thaliana* correspondingly targets the cleavage of *MYB33* and *MYB101* transcripts, resulting in seed insensitivity to ABA signaling [28]. Under light-induced conditions, miR163 can promote seed germination by targeting *PXMT1* [29]. Cumulative evidence shows that miRNA from various plants is involved in seed dormancy and germination processes, and miRNA-regulated gene expression is an important pathway for plants to regulate seed dormancy and germination at the molecular level.

At present, high-throughput small RNA sequencing and degradome sequencing technologies are powerful and useful tools for studying miRNA functions at various developmental stages. The combination of both technologies has effectively been used to identify numerous miRNAs and their target genes in numerous plant species [30]. A study identified differentially expressed miRNAs during the chilling-induced dormancy release of tree peony (*Paeonia suffruticosa* Andrews) [31]. Another investigation recognized microRNAs associated with the dormancy release during cold storage of *Lilium pumilum* Bulbs [32]. Likely, miRNAs responsive to ABA and GA in maize (*Zea mays*) embryos were observed during seed germination [33]. Currently, the exploration of potato miRNAs is primarily concentrated on different biotic and abiotic stresses, including drought stress [34], low nitrogen stress [35], alkali stress [36], cadmium stress [37], potato virus A (PVA) [38], light response [39], and low-temperature stress [40]. However, despite a lot of evidence related to tuber dormancy and sprouting, as well as their related mechanisms in different crop species, potato tuber dormancy and sprouting are still a question for researchers.

This is the very first study to determine the miRNAs related to potato tuber dormancy release and sprouting. We use the potato (*S. tuberosum*) cultivar ‘Atlantic’ as material in this study, which is medium-maturing with a growth period of ~90 days and a moderate dormancy period. Moreover, we utilized high-throughput small RNA (sRNA) sequencing combined with degradome sequencing technologies to investigate miRNAs and their target genes involved in potato tuber dormancy and sprouting, as well as their regulatory functions. Furthermore, we identified a novel miRNA (novel_220) named stu-miR319c that is associated with tuber dormancy release. The role of stu-miR319c and its target genes, *StTCP26* and *StTCP27*, in regulating tuber dormancy release were investigated using transient co-expression systems, overexpression, and silencing transgenic technology, and the possible mechanisms of the miR319c participating in JA regulatory pathway were explored. This study provides a fresh perspective for researchers to understand the function of novel microRNAs (stu-miR319c) in potato tuber dormancy and sprouting, which will benefit future molecular breeding.

## Results

### Determination of three periods of potato tuber dormancy release process

**Figure 1:**
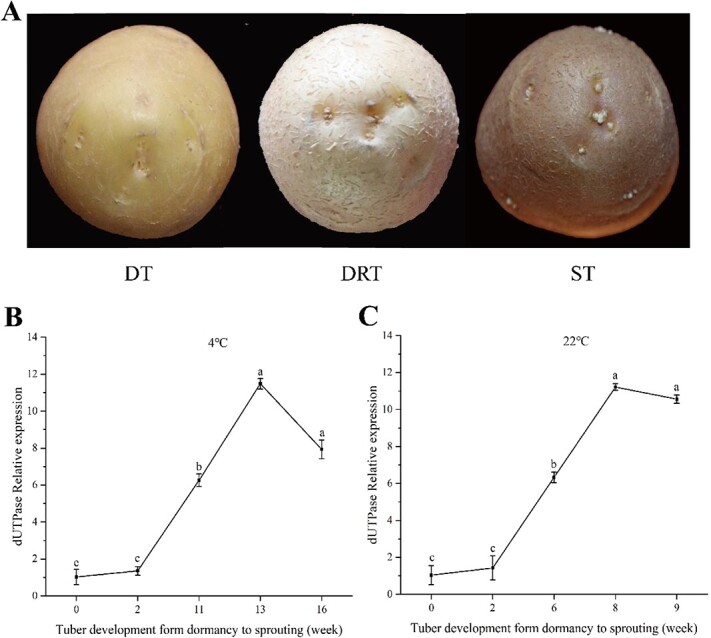
Morphological changes and expression profile of dUTPase gene during tuber dormancy release progression in potato. (A) Morphological changes of bud eye during tuber dormancy release in potato. (B) dUTPase gene expression profile of tuber stored at 4°C. (C) dUTPase gene expression profile of tuber stored at 22°C. The data represented mean ± standard deviation. Lowercase letters indicate the significant difference (at *P* <0.05) based on Tukey’s test.

The period of potato tuber dormancy and sprouting was determined according to morphological characteristics (Fig. 1A). The expression pattern of dUTPase (deoxyuridine triphosphatase) genes associated with DNA synthesis and cell division was detected by quantitative real-time polymerase chain reaction (qRT-PCR) to determine the period of tuber dormancy release (DRT), and the results showed that dUTPase gene expression was maximum at 13th and 8th weeks under storage conditions at 4 and 22°C, respectively (Fig. 1B and C). Finally, through the changes in morphological characteristics and gene expression, tubers from 0, 13, and 16 weeks of storage at 4°C and 0, 8, and 9 weeks of storage at 22°C were selected as dormant tubers (DT), dormant released tubers (no visible shoots, DRT), and tubers (2–3 mm visible shoots, ST) for sRNA high-throughput sequencing and analysis.

### miRNA sequencing of the tuber dormancy release process

miRNA sequencing was utilized to discover miRNAs associated with the processes of tubers from dormancy to sprouting stage samples at 4 and 22°C. A total of 286 400 300 raw reads were collected from 18 libraries from the dormancy release processes of potatoes (Table S1). After eliminating the adaptors and low-quality reads, 275 826 291 clean reads were obtained and documented in Table S1. Unique readings were obtained by eliminating repeated sequences of clean reads, and the frequency of length distribution was counted. The results showed that the sRNA length of most clean reads was 21–24 nt, and the sRNA length of 24 nt was the most abundant (Fig. S1). The sRNAs after length screening were mapped to the reference genome, and the data showed that an average of 89.09% of clean reads were perfectly mapped to the potato genome (Table S2). Each unique sRNA was annotated based on a priority order that includes known miRNA, ribosomal RNA (rRNA), transfer RNA (tRNA), small nuclear RNA (snRNA), small nucleolar RNA (snoRNA), repeat, small interfering RNAs originated from natural antisense transcripts (NAT-siRNA), gene, novel miRNA, and trans-acting small interfering RNA (ta-siRNA). Approximately 0.98 and 1.34% of the sRNA numbers were recognized as known and novel miRNAs (Table S3). About 0.06 and 0.07% of the sRNA types were recognized as known and novel miRNAs (Table S4).

### The expression characteristics of miRNAs in potato tuber dormancy release

By investigating the miRNA sequencing data, we identified 396 miRNAs in the six libraries of tubers, including 204 known and 192 novel miRNAs (Table S5 and S6). As shown by the flower plot, the six libraries collectively contained 123 known and 177 novel miRNAs, and each library contained a range of 152–171 known miRNAs and 183–186 novel miRNAs (Fig. S2A and B). The identified miRNAs varied in length from 20 to 25 nt, with miRNAs of 21 and 24 nt being the most common among known and novel miRNAs, respectively (Fig. S2C). The 5′ position of these miRNAs had a strong preference for uracil (U) and adenine (A) (Fig. S3A and B), which are related to the fact that most miRNAs can bind to AGO1 to play a specific function. Furthermore, the family analysis of 157 miRNA precursors revealed that 94 miRNA precursors and 132 conserved mature miRNAs belong to 46 miRNA families, and 72 nonconserved miRNAs are presumably specific to potatoes (Fig. S2D; Table S7).

### Identification of the temperature-induced tuber dormancy release process–related miRNAs

**Figure 2:**
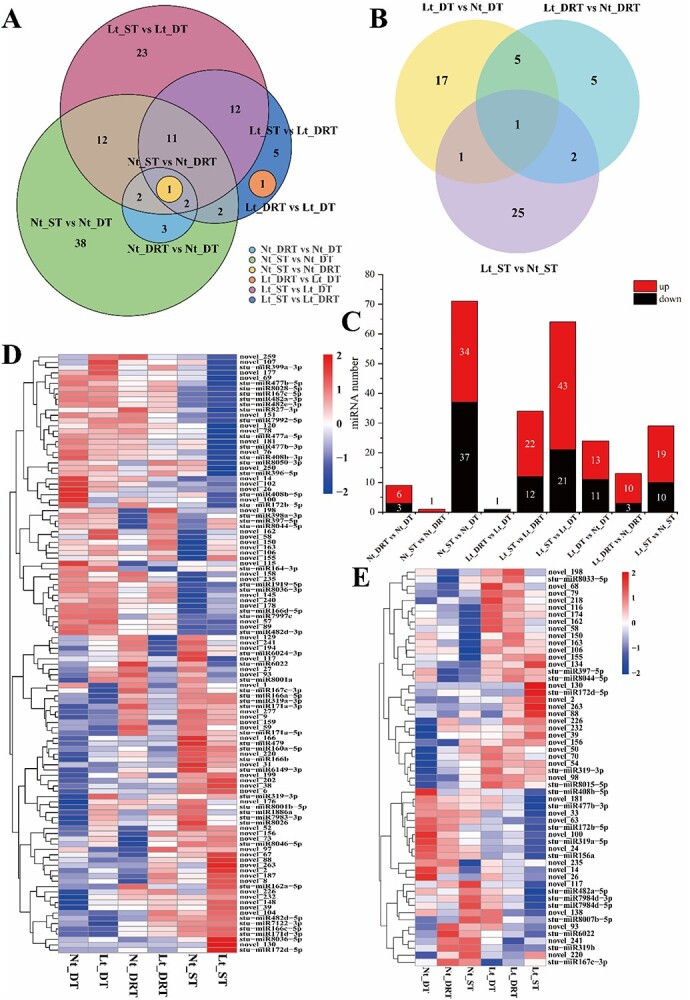
Identification of differentially expressed miRNAs in tubers. (A) Venn diagram displaying the number of DE-miRNAs involved in regulating tuber dormancy release. (B) Venn diagram displaying the number of DE-miRNAs involved in regulating tuber dormancy release by temperature. (C) Number of DE-miRNAs in each comparison sample. (D) Expression profile and clustering of DE-miRNAs involved in regulating tuber dormancy release. (E) Expression profile and clustering of DE-miRNAs involved in regulating tuber dormancy release by temperature. *Nt_ = normal temperature (22°C), Lt_ = low temperature (4°C), DT = dormant tuber, DRT = dormant released tuber, ST = spouting tuber.

To investigate the differentially expressed miRNAs (DE-miRNAs) involved in regulating tuber dormancy release, we compared the expression of miRNAs during the dormancy to sprouting period [Nt_DRT vs Nt_DT, Nt_ST vs Nt_DRT, Nt_ST vs Nt_DT, Lt_DRT vs Lt_DT, Lt_ST vs Lt_DRT, Lt_ST vs Lt_DT; in which Nt_ represents normal temperature (22°C), and Lt_ represents low temperature (4°C)]. The results showed that a total of 113 DE-miRNAs were screened, including 49 known and 64 novel miRNAs (Fig. 2A, C, and D). Under normal temperature conditions, there are eight DE-miRNAs in Nt_DT, Nt_DRT, and Nt_ST three periods. Five miRNAs were up-regulated, with novel_9, novel_220, and stu-miR171a-5p significantly differentially expressed and highly expressed. Three down-regulated genes were novel_26, stu-miR398a-3p, and stu-miR166d-5p. Under low-temperature conditions, there are 26 DE-miRNAs in Lt_DT, Lt_DRT, and Lt_ST three periods. Nineteen miRNAs were up-regulated, with stu-miR166a-5p, stu-miR319a-3p, novel_220, novel_6, novel_159, and novel_2 significantly differentially expressed and highly expressed. Seven miRNAs were down-regulated, with stu-miR477b-5p, stu-miR482a-3p, and stu-miR482e-3p significantly differentially expressed and highly expressed. The Venn diagram found an up-regulated and highly expressed miRNA novel_220, differentially expressed in tuber dormancy release under normal- and low-temperature conditions (Fig. 2A; Table S8).

To investigate the DE-miRNAs during temperature-induced tuber dormancy release, we compared the expression of miRNAs during the dormancy to sprouting period at different temperatures (Lt_DT vs Nt_DT, Lt_DRT vs Nt_DRT, Lt_ST vs Nt_ST). The results showed that 56 DE-miRNAs regulated by temperature during tuber dormancy release were screened, including 18 known and 38 novel miRNAs (Fig. 2B, C, and E). The miRNAs with large fold difference and high expression were novel_220, stu-miR6022, stu-miR319a-5p, novel_14, and novel_26 in Lt_DT vs Nt_DT period, and were novel_54, stu-miR319-3p, stu-miR319b, and stu-miR167c-3p in Lt_DRT vs Nt_DRT period, and were novel_88, novel_263, stu-miR8044-5p, stu-miR7984d-3p, stu-miR7984d-5p, and stu-miR482a-5p in Lt_ST vs Nt_ST period. The differential Venn diagram found a down-regulated miRNA (novel_63), regulated by temperature under all three periods (Fig. 2B; Table S8).

The expression of miRNAs discovered through deep sequencing was further examined using qRT-PCR for nine miRNAs (Fig. S4). Only one miRNA (stu-miR166b) showed expression patterns that did not match the sequencing data when analyzed using qRT-PCR. The rest of the qRT-PCR results aligned with the sequencing data. It shows that sequencing data accurately reflect the expression levels of miRNAs in the six libraries.

### Identification and analysis of miRNA targets through degradome sequencing

In plants, miRNAs typically function as leading RNAs and stimulate the cleavage of target messenger RNAs (mRNAs). Thus, the elucidation of miRNA regulatory functions is inseparable from the identification of miRNA-regulated target genes. With a set score of ≤2.5, PSROBOT software was utilized to predict 313 target genes for 138 miRNAs, including 86 known miRNAs and 52 novel miRNAs (Table S9). To validate the miRNA–target interaction *in vivo*, degradome sequencing was conducted to identify cleavage mRNA using PAREsnip [41]. In total, 821 target genes of 202 miRNAs were discovered as splicing transcripts, including 548 targets of 125 known miRNAs and 273 targets of 77 novel miRNAs (Table S10). Sixty-five target genes showed overlap between PSROBOT (score ≤2.5) and PARESNIP (category ≤2), and some existing genes were unpredicted in PSROBOT (Tables S9 and S10). This proved that degradome sequencing can predict more possible and credible target genes.

Analysis of these target genes revealed that miRNAs are engaged in several pathways. Some of the target genes of miRNA were identified as TFs, such as growth-regulating factor GRF (targeted by stu-miR396-5p, novel_3, novel_16), auxin response factor ARF (targeted by stu-miR160a-5p, stu-miR167c-5p, stu-miR8047, novel_130), ET-responsive TF (AP2 and ERF, targeted by stu-miR172b-3p, stu-miR172c-3p, stu-miR172d-3p, stu-miR3627-3p, novel_105), and TF TCP (targeted by stu-miR319-3p, novel_220, novel_1, novel_6). On the other hand, several target genes identified are associated with transport and catabolic processes, such as autophagy-related protein ATG16 (targeted by stu-miR167a-3p) and uridine diphosphate (UDP)-apiose/xylose synthase (targeted by stu-miR8044-5p). Finally, certain genes were identified as other functional proteins, such as acetyl-CoA carboxylase biotin carboxyl carrier protein (targeted by stu-miR167b-3p and stu-miR399j-5p) and ABC transporter protein (targeted by stu-miR391-3p, stu-miR172c-3p, novel_71) (Table S10). Gene Ontology (GO) analysis discovered that these target genes were enriched in biological processes such as cell differentiation (GO:0030154), seed development (GO:0048316), seed germination (GO:0009845), embryo development ending in seed dormancy (GO:0009793), and response to hormones including ET, ABA, GA, JA, IAA, CTK, SA, and BR (GO:0009725, GO:000972, GO:0009737, GO:0009739, GO:0009753, GO:0009735, GO:0009733, GO:0009751, GO:0009742), respectively (Fig. S5A; Table S11). These target genes play essential roles in gene regulation and plant growth and development.

### miRNAs involved in phytohormone signaling during tuber dormancy release and sprouting

**Figure 3:**
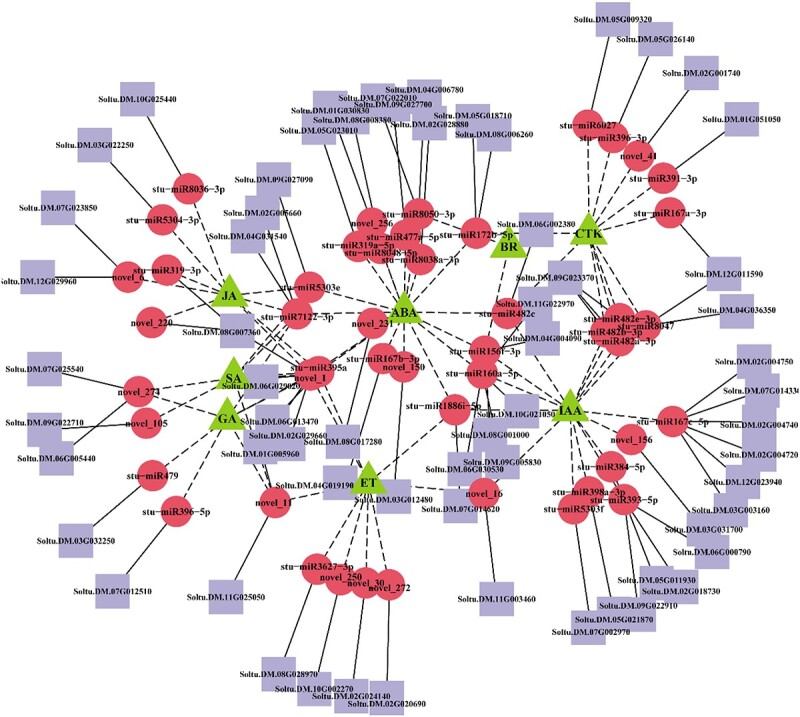
Regulatory network of phytohormone-related miRNAs involved in tuber dormancy release and sprouting. The miRNA–mRNA interactions visualized as a network using cytoscape; the triangle (green) represents hormone, the circle (red) represents miRNA, and the rectangle (purple) represents mRNA.

Hormones are crucial in tuber dormancy release and sprouting processes. KEGG (Kyoto Encyclopedia of Genes and Genomes) and GO analyses were performed for the target gene candidates (Fig. S5B; Table S12 and S13). Sixty-three target genes and 48 miRNAs were predicted to be involved in plant hormone signaling pathways, including 15 novel miRNAs and 33 known miRNAs (Fig. 3). Eighteen, 7, 12, 8, 9, 9, 2, and 7 miRNAs were identified to be involved in ABA, GA3, IAA, CTK, JA, ET, BR, and SA signal transduction, respectively. Among them, stu-miR5303 is involved in the four hormone pathways of ABA, IAA, JA, and SA. stu-miR167 is involved in the three hormone pathways of ABA, IAA, and CTK. stu-miR156 is involved in the three hormone pathways of ABA, IAA, and BR. stu-miR160 is involved in the three hormone pathways of ABA, IAA, and ET. stu-miR395 and stu-miR7122 are involved in the three hormone pathways of ABA, JA, and SA. stu-miR172 and stu-miR482 are involved in the ABA and CTK hormone pathways. stu-miR8050 is involved in the ABA and BR hormone pathways, while stu-miR319 is involved in the ABA hormone pathway and is also predicted to be associated with the JA pathway. Most of these miRNAs are involved in at least two plant hormonal pathways, and it is speculated that the control of the tuber dormancy release process involves crosstalk among eight plant hormones.

### Stu-miR319c characteristics and its target genes

**Figure 4:**
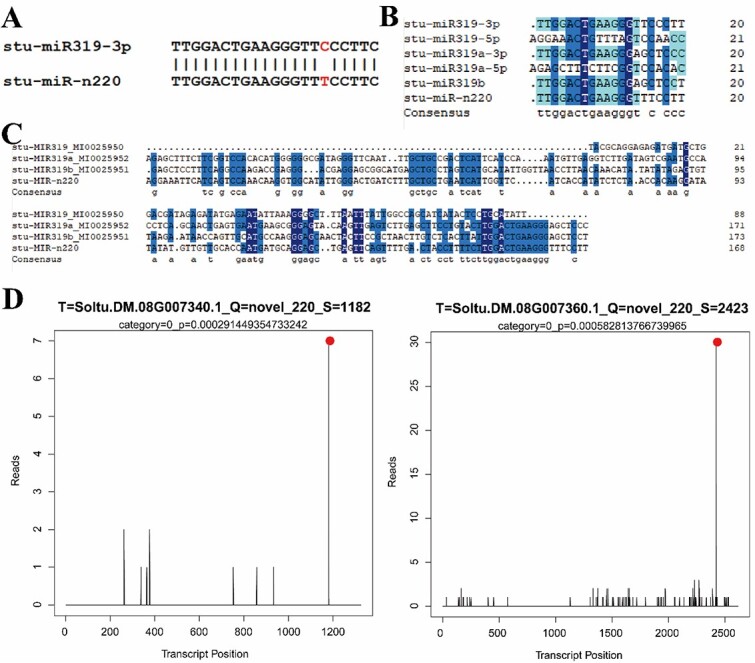
Sequence alignment and target gene analysis of miRn220. (A) Homologous sequence alignment of stu-miRn220. (B) Mature body sequence alignment of stu-miRn220 and stu-miR319 family members. (C) Precursor sequence alignment of stu-miRn220 and stu-miR319 family members. (D) T-plot of target genes of stu-miR319. The red solid dot indicates the cleavage site of the target gene.

As shown by the Venn diagram of DE-miRNAs (Fig. 2A), only one miRNA was differentially expressed in the three periods of dormancy, dormancy release, and sprouting under normal temperature. This miRNA, novel_220, was an up-regulated and highly expressed gene (Fig. S6), which was regarded as the main focus of our subsequent research. To find the homologous miRNA of stu-miRn220, the mature body sequence was submitted to the miRBase database for comparison, and the results showed that the mature body sequences of stu-miRn220 and stu-miR319-3p differed by only one base, so stu-miRn220 belonged to the members of the stu-miR319 family and was named miR319c based on the already existing members of the family (Fig. 4A and B).

miRNA has been demonstrated to regulate gene expression by direct cleavage and translation suppression in the nucleus [42, 43]. Analysis of the degradome data identified six target genes of stu-miR319c (Table S14), of which *StTCP26* (Soltu.DM.08G007340) and *StTCP27* (Soltu.DM.08G007360) were significantly cleaved at 1182 and 2423 bp, respectively. The target genes *StTCP26* and *StTCP27* are classified under category 0, displaying a single peak, with the cleavage site mediated by stu-miR319c overlapping with the peak (Fig. 4D).

**Figure 5:**
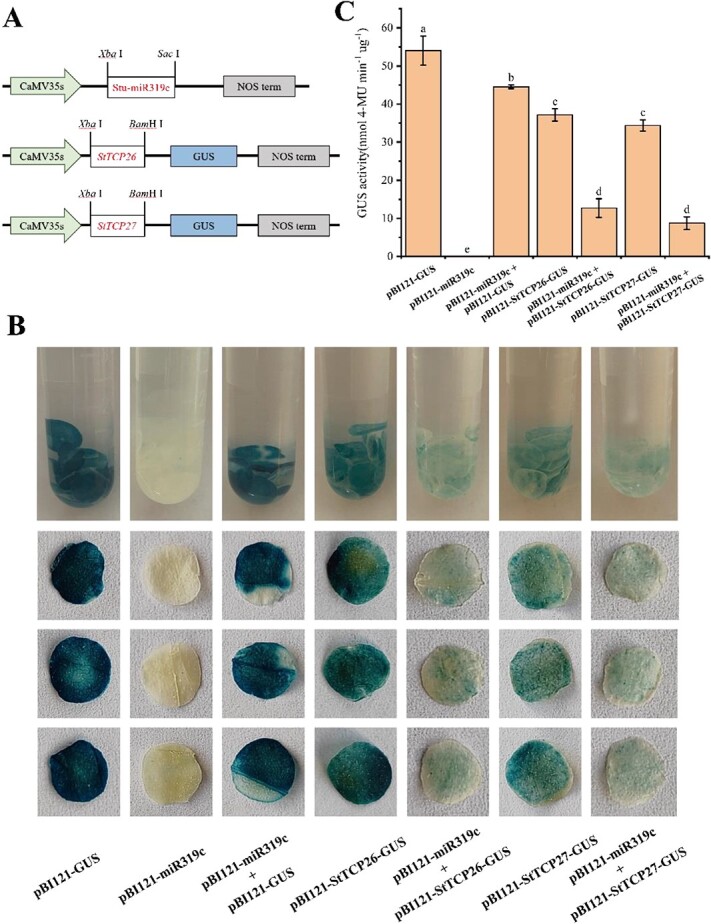
Interaction between stu-miR319c and target *StTCP* by cotransformation in tobacco leaves. (A) Vector construction for cotransformation of stu-miR319c and *StTCP* in tobacco leaves. (B) The different recombinant vectors were transferred into tobacco leaves by *Agrobacterium* strain GV3101, and the GUS phenotype was observed by histochemical staining. (C) GUS enzyme detection. The data represented mean ± standard deviation. Lowercase letters indicate the significant difference (at *P* <0.05) based on Tukey’s test.

A cotransformation experiment was conducted in tobacco (*Nicotiana benthamiana*) leaves to investigate the degradation of StTCP26 and StTCP27 by stu-miR319c. The experiment utilized the vector pBI121 with the GUS reporter gene, and the phenotype of leaves infected with pBI121 was evaluated by GUS staining technology (Fig. 5). The leaves injected with pBI121-*StTCP26-*GUS and pBI121-*StTCP27-*GUS, in which *StTCP26 and StTCP27* were linked ahead of the GUS reporter gene, displayed a comparable appearance to the control. Tobacco leaves were injected with pBI121-miR319c bacteriophage, leading to a lack of GUS phenotype. To eliminate the possibility of pBI121-miR319c inhibiting the empty vector, we injected leaves with cotransformed pBI121-GUS and pBI121-miR319c, and it was clearly observed that the GUS phenotype was unaffected. GUS histochemical staining was notably hindered in tobacco leaves transforming with pBI121-miR319c mixed with pBI121-*StTCP26*-GUS and pBI121-*StTCP27*-GUS, respectively (Fig. 5A and B). GUS activity analysis aligned well with the histochemical results (Fig. 5C). The results confirmed that stu-miR319c effectively targets *StTCP26* and *StTCP27*, leading to a considerable suppression of their expression.

**Figure 6:**
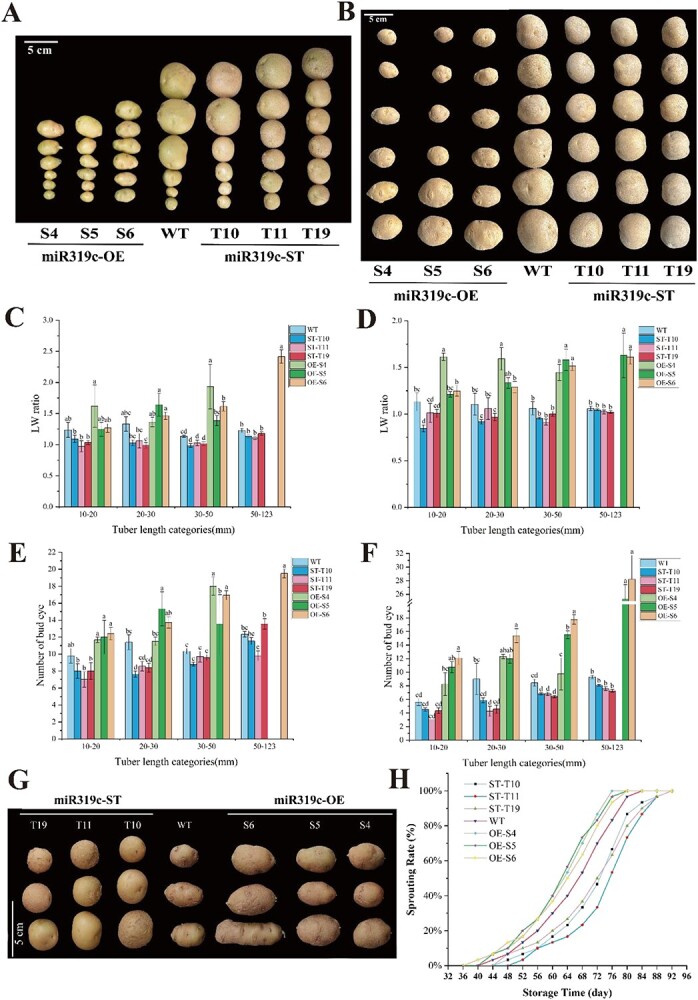
Morphological traits and sprouting analysis of stu-miR319c transgenic tubers. (A, B) Tuber shape (A: first-year tuber, B: second-year tuber). (C, D) Tuber length–width ratio (C: first-year tuber, D: second-year tuber). (E, F) Number of bud eyes (E: first-year tuber, F: second-year tuber). (G) Sprouting observation. (H) Sprouting rate of stu-miR319c transgenic and WT tubers. All data represented mean ± standard deviation. Lowercase letters indicate the significant difference (at *P* <0.05) based on Tukey’s test.

### Subcellular localization of StTCP and expression pattern of stu-miR319c/StTCP in various tissues

After the construction of subcellular localized vectors (StTCP26-EGFP, StTCP27-EGFP) and empty vectors (EGFP), they were infiltrated into tobacco leaves by *Agrobacterium*-mediated transformation, and the transient expression of the fusion proteins was observed using laser confocal scanning electron microscopy. The results showed that StTCP26-EGFP and StTCP27-EGFP recombinant fusion proteins had strong EGFP green signals only on the nucleus (Fig. S7A), which indicates that StTCP26 and StTCP27 have typical TF characteristics.

The stu-miR319c expression was significantly increased in the tuber sprouting period, as confirmed by both miRNA sequencing data and qRT-PCR validation (Fig. 2; Table S8; Fig. S4). To confirm the expression pattern of stu-miR319c targeting *StTCP26* and *StTCP27,* qRT-PCR was used for the study. The results showed that from dormancy to sprouting of the tubers, the *StTCP26* and *StTCP27* were first increased and then decreased (Fig. S7B). Overall, it is feasible to speculate that stu-miR319c/StTCP module may have a significant regulatory function during the dormancy release process of tubers. Furthermore, we detected the expression levels of stu-miR319c as well as the target genes *StTCP26* and *StTCP27* in seven distinct tissues. The results showed that stu-miR319c had the highest expression in the tuber, and was 51-folds in other low-expression tissues, particularly in leaves. The expression levels of *StTCP26* and *StTCP27* were the lowest in the tuber and the most abundant in the terminal bud and lobed leaf, and their expression pattern was inversely linked with stu-miR319c in the tuber (Fig. S7C). The expression patterns of stu-miR319c/StTCP in different tissues offer valuable insights for investigating their biological roles in potato.

### Morphological characteristics of stu-miR319c transgenic potato plants

To investigate whether stu-miR319c is involved in the biological function of tuber dormancy release. The miR319c was overexpressed in the wild-type (WT) potato cultivar ‘Atlantic’ under the control of the 35S promoter (Fig. S8). qRT-PCR results demonstrated the reduced expression of *StTCP26* and *StTCP27* in three miR319c overexpressing (miR319c-OE) transgenic lines (Fig. S9A). At the same time, we utilized the short tandem target mimic (STTM) technology to reduce the miR319c expression level (Fig. S8). The qRT-PCR analysis demonstrated effective upregulation of both *StTCP26* and *StTCP27* expression in miR319c-ST transgenic lines (Fig. S9A). The above results indicate that miR319c negatively regulates the expression of the target genes *StTCP26* and *StTCP27*.

Previous research has demonstrated that miR319 family genes play a role in regulating plant growth and development [44]. In this experiment, phenotypic changes were also found in overexpressing (miR319c-OE) and knockdown expression (miR319c-ST) transgenic potato lines. We observed the shape and compared the length–width ratio of the leaves (Fig. S9B and C). The results showed that the length–width ratio of leaves in miR319c-ST lines was lower than that in nontransgenic plants (WT), and the leaves became rounder and flatter. Among them, the biggest difference was the ST-T11 transgenic line. In miR319c-OE lines, the length–width ratio of leaves was higher than that in WT plants, the leaves became narrower and wrinkled, and the symmetry of some leaves was also broken. Among them, the biggest difference was in the OE-S4 transgenic line. The above results indicate that stu-miR319c also regulates the shape of leaves.

To further study the role of stu-miR319c in the regulation of potato morphology, the transgenic and nontransgenic plants were subcultured on Murashige & Skoog (MS) medium for 1 month, and then, we observed and compared their morphological characteristics and analyzed the differences (Fig. S9D). The results showed that the mean values of plant height, root length, fresh weight, and root–shoot ratio of miR319c-ST transgenic lines were 0.37, 1.87, 0.37, and 2.50 times higher than those of WT plants, respectively. However, no significant differences were observed between the miR319c-OE plants and WT for plant height, root length, fresh weight, root fresh weight, and root–shoot ratio (Fig. S9D; Table S15). The above results indicated that the growth of plant roots was significantly promoted and plant height was inhibited after miR319c gene interference. However, there was no significant change in the plants after overexpression of the miR319c gene. It is thought that miR319 has a crucial regulatory role in potato growth and development.

The morphological traits of stu-miR319c transgenic tubers were also analyzed during this study. According to the Technical Code for Evaluating Germplasm Resources of Potato (*S. tuberosum* L.) (NY/T 1303-2007), to understand, the tuber shape is divided into 17 types (flat circular, round, ovate, obovate, flat elliptical, elliptical, oblong, etc.), the number of bud eyes is divided into three types [less (the number of bud eyes <7), medium (7 ≤ the number of bud eyes ≤12), and more (the number of bud eyes >12)], and the smoothness of the potato skin is divided into three types [smooth (smooth skin without mesh), medium (smoother skin with mesh), and rough (rough skin with heavy hemp)]. In addition to the QTL (quantitative trait locus) analysis of tuber shape and bud depth by Prashar *et al*. [45], we can understand the identification of our study potato shape and data analysis for mapping. The tuber shapes of miR319c transgenic and nontransgenic tubers in 2 years were observed, and the differences in tuber length–width ratio and bud eye number were also analyzed (Fig. 6A–F). The results showed that compared with WT tubers, the length–width ratio of miR319c-ST transgenic tubers decreased, and the tuber shape became rounder to oblate, with rougher epidermis and fewer bud eyes. On the contrary, the length–width ratio of miR319c-OE transgenic tubers increased, and the tuber shape changed from round to oval, with a smooth epidermis and more bud eyes. The above results indicate that stu-miR319c regulates the shape of tubers, the smoothness of tuber skin, and the number of bud eyes. The results of the 2-year tuber traits were consistent, indicating that this trait was a stable inheritance, and it was thought that stu-miR319 played a crucial regulatory role in tuber growth and evolution.

### stu-miR319c promotes sprouting of potato tuber

To elucidate the role of miR319c in potato dormancy release and sprouting, the sprouting of tubers stored in darkness at room temperature (22 ± 1°C) was observed and recorded. The results showed that when the dormancy of WT tubers was released, and white spots appeared at the bud eye, the tubers of miR319c-OE lines had already sprouted or had longer buds than the WT tubers. At the same time, the tubers of miR319c-ST lines had delayed germination compared to WT and were still in the dormancy period (Fig. 6G). Sprouting statistics showed that the first visible sprouts appeared on WT tubers after 44 days, and 100% sprouting was reached after 84 days. The tubers of miR319c-OE lines appeared the first visible sprouts on average at 41 days, and 100% sprouting was reached after 79 days which was 5 days earlier than that of the WT. The tubers of miR319c-ST lines appeared the first visible sprouts on average at 49 days, and 100% sprouting was reached after 92 days, which was delayed 8 days than the WT (Fig. 6H). The above results suggest that stu-miR319c can promote the dormancy release and sprouting of potato tuber.

### Jasmonic acid accelerates the sprouting of potato tuber

**Figure 7:**
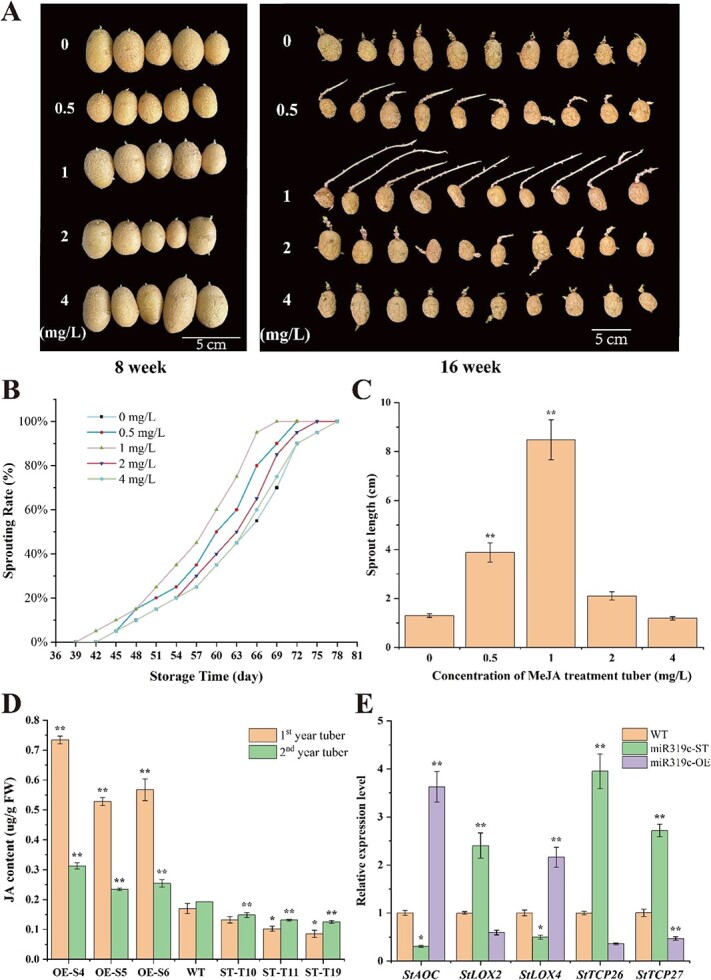
miR319c mediates the JA pathway to promote tuber sprouting. (A) Changes in morphology of MeJA-treated tubers after 8 and 16 weeks. (B) Sprouting rate of MeJA-treated tubers. (C) Statistical results of sprout length after 16 weeks. (D) JA content in miR319c transgenic tubers. (E) Expression levels of JA pathway–related genes in miR319c transgenic tubers. The data represented mean ± standard deviation. *P*-values (^*^P <0.05; ^**^P <0.01) were calculated by the Dunnett test.

JA signaling plays a role in multiple physiological responses in seed plants [46]. Moreover, previous studies reported that the miR319/TCP module has a role in various developmental processes, primarily via influencing JA synthetic genes and the endogenous JA level [47]. In this experiment, the WT tubers were treated with different concentrations of exogenous MeJA and then stored in the dark at room temperature (22 ± 1°C) to observe sprouting, in order to further explore the role of JA in dormancy release and sprouting of tuber. From dose trials using a range of MeJA concentrations (0, 0.5, 1, 2, and 4 mg/L), we observed and statistically analyzed that 1 mg/L was the most effective concentration to promote the dormancy release of tubers and significantly improved the sprouting rate of tubers, with complete germinating occurring ~9 days earlier than the control group. Furthermore, treatment with 4 mg/L MeJA had no effect on tuber sprouting (Fig. 7A and B). Correspondingly, the length of buds was measured after 16 weeks of MeJA treatment. The results showed that the average sprout length was 1.3 cm in the control, and the average sprout length was 8.0 cm under 1 mg/L MeJA treatment, which was six times that of the control. The average sprout length was 1.2 cm under 4 mg/L MeJA treatment, which was similar to that of the control (Fig. 7C). The above results indicated that JA of low concentration significantly broke dormancy and enhanced the sprouting ability of potato tubers.

### Jasmonic acid is involved in miR319c-regulated dormancy release and sprouting of tuber

To further explore whether the miR319c affects the dormancy release of tubers by participating in the JA pathway, we detected JA content in miR319c transgenic tubers. As shown in the 2-year results, compared with WT tubers, JA content was significantly increased in miR319c-OE line tubers and significantly decreased in miR319c-ST line tubers (Fig. 7D). The results indicated that stu-miR319c positively regulated the synthesis of JA in potato tuber.

13-LOX (13-lipoxygenase) catalyzes the formation of 13-HPOT (13-hydroperoxy-linoleic acid) from the JA synthesis precursor, and AOC (allene oxide cyclase) catalyzes the formation of oxophytodienoic acid (OPDA) from 13-HPOT, both of which are essential enzymes in the JA synthesis pathway and involved in plant growth and development [48, 49]. According to the transcriptome data, it was found that *StAOC* and *StLOX* were differentially expressed during tuber dormancy to sprouting [19]. The expression levels of *StAOC* (Soltu.DM.02G025590), *StLOX2* (Soltu.DM.08G005440), *StLOX4* (Soltu.DM.03G037120), *TCP26*, and *TCP27* genes were assessed by qRT-PCR in transgenic tubers during dormancy release periods. The qRT-PCR analysis results showed that compared with WT, the expression levels of *StAOC* and *StLOX4* genes were dramatically increased, while the expression level of *StLOX2* was significantly decreased in miR319c-OE transgenic tubers. In contrast, the expression levels of *StAOC* and *StLOX4* genes were considerably decreased, and the expression level of *StLOX2* was increased in miR319c-ST transgenic tubers (Fig. 7E). The results indicated that the miR319c may regulated JA production by affecting transcripts of genes involved in JA synthesis. The expression levels of *StTCP26* and *StTCP27* were significantly lower in miR319c-OE tubers compared with WT tubers and significantly higher in miR319c-ST tubers (Fig. 7E). This result further verified the splicing effect of miR319c with *StTCP26* and *StTCP27* genes. Collectively, the findings indicated that Stu-miR319c affected tuber dormancy release possibly by inhibiting the expression of the target genes *StTCP26* and *StTCP27* and participating in the JA pathway.

## Discussion

Potato (*S. tuberosum* L.) is one of the most significant staple foods, which is the fourth largest crop and third most consumed crop around the world. However, tuber dormancy and sprouting are the most important features of tuber physiological properties throughout their life cycle, determining potato development and yield [15]. In view of the enormous market demand for potatoes, it is very critical to develop such genetic mechanisms to control the storage (dormancy) and sprouting period of potato tubers [50]. In this study, we identified a novel miRNA (miR319c) through genome-wide analysis of noncoding RNA, which plays a significant role in potato tuber dormancy release pathways.

### Differentially expressed microRNAs during the dormancy to sprouting process of potato tubers

Over the last 10 years, significant focus has been directed toward studying potato miRNA, which plays a role in various biological processes including alkali stress [36], disease resistance [51], leaf and flower development [52], and tuber development [53]. However, the role and function of miRNAs during potato tuber dormancy in the sprouting process are less appreciated. Therefore, more miRNAs related to the tuber dormancy-sprouting transition can be discovered by high-throughput sequencing and confirmed using different approaches.

In this study, a total of 204 known and 192 novel miRNAs were identified using high-throughput sequencing technology that involved tubers at 4 and 22°C from dormancy to the sprouting stage (Tables S5 and S6). It was also found that the identified sRNAs were predominantly 24 nt in length, followed by 22 and 21 nt (Fig. S1). The miRNAs with lengths of 21 and 24 nt were the most abundant types of known and novel miRNAs, respectively (Fig. 2C). Similar length distributions of sRNAs and miRNAs were also observed in previous studies on potato [51], as well as other plant species including *Arabidopsis* [54], tomato [55], and tea plant [47]. It is emphasized here that more miRNA families belonging to conserved miRNAs were identified in this study (Tables S5 and S6) compared to previous studies [36, 56] on potatoes, presumably due to deeper sequencing data coupled with the newest miRbase database, or perhaps more miRNAs are involved in tuber dormancy and sprouting processes. This greatly broadens our comprehension of the biological roles of miRNAs during potato tuber dormancy release.

Exploring the expression profile of some key miRNAs is crucial for comprehending the mechanism of tuber from dormancy to sprouting in potato. Our analysis identified that 113 miRNAs from potato tubers were differentially regulated from dormancy to sprouting, including 49 known miRNAs (Fig. 2A, C, and D). According to reports, the miR166/HD-ZIP III module can control cell differentiation in the root and shoot apical meristems of *Arabidopsis* [57]. This study discovered four members of the stu-miR166 family (miR166a-5p, miR166b, miR166c-5p, and miR166d-5p) that are involved in tuber dormancy and sprouting. During the transition from dormancy to sprouting, miR166a-5p, miR166b, and miR166c-5p were up-regulated, but miR166d-5p was down-regulated (Fig. 2D; Table S8). It is hypothesized that stu-miR166 family members are involved in different pathways regulating tuber dormancy release, which showed similar mechanisms in the functions of microRNA. Previous research elaborated that the miR393-mediated negative regulation of *OsTIR1* and *OsAFB2* auxin receptor genes leads to the dormancy of rice (*Oryza sativa* L.) seeds [58]. Parallel results were described in another report that miR393 expression was high and up-regulated in barley (*Hordeum vulgare* L.) seed germination [59]. However, the miR393-3p and miR393-5p members of the stu-miR393 family found in this study had extremely low expression levels during the tuber dormancy to germination process (Table S8), implying that this could be attributable to species-specific miRNA expression patterns.

Furthermore, a total of 56 DE-miRNAs, including 18 known miRNAs, were differentially regulated by temperature during dormancy release (Fig. 2B, C, and E). Three members of the stu-miR319 family (miR319-3p, miR319a-5p, and miR319b) were responsive to cold temperatures (Fig. 2E). Their homologs are also known to be cold-responsive in other species. Such as miR319c in *Arabidopsis* specifically responds to low-temperature stress, showing a notable increase in miR319c accumulation following treatment at 0°C [60]. The expression of miR319 in maize was down-regulated after low-temperature stress [61]. Our study demonstrated that stu-miR397 expression increased during the sprouting period of tubers stored at 4°C (Table S8), which was similar to the results of up-regulation of miR397 expression in *Arabidopsis* in response to low-temperature signaling [62]. Cold treatment decreased stu-miR408b-5p expression in potato dormancy (Table S8) while enhancing miR408 expression in maize [61]. Furthermore, our study revealed that stu-miR167c-3p exhibited a decrease in expression levels when subjected to cold treatment throughout the stages of tuber dormancy, dormancy release, and sprouting (Fig. 2E; Table S8). Additionally, this down-regulation was observed not only in potatoes but also in rice [63] and soybean (*Glycine max*) [64]. Nevertheless, it exhibited an increase in expression levels in tomato (*S. lycopersicum*) [65] and *Arabidopsis* [66]. The study emphasizes the complexity of miRNA regulation of tuber dormancy release and highlights the divergence of miRNA expression patterns across species and experimental settings.

### MicroRNAs involved in phytohormone signaling during tuber dormancy release and sprouting

The dynamic changes of endogenous hormones, such as GA, IAA, ABA, ET, CTK, and JA, significantly affect the growth status during tuber dormancy release progress [67]. miRNAs have a crucial role in regulating plant hormone response pathways through influencing their metabolism, distribution, and perception [68]. We performed hormonal pathway analysis on target genes obtained by degradome sequencing (Fig. S5B; Table S12), and 63 target genes and 48 miRNAs were predicted to be involved in plant hormone (ABA, GA3, IAA, CTK, JA, ET, BR, and SA) signaling pathways, including 15 novel miRNAs and 33 known miRNAs (Fig. 3). Since one miRNA regulates multiple target genes and one target gene can also be regulated by multiple miRNAs [69], most of the miRNAs involved in the plant hormone signaling pathway are predicted to be involved in at least two phytohormone pathways (Fig. 3; Table S13), speculating that the regulation of tubers from dormancy to sprouting involves the interaction between plant hormones. In a former report, it was discovered that under drought conditions, miR159 in *Arabidopsis* seeds responded to ABA and increased its expression, while the levels of *MYB33* and *MYB101* declined. Furthermore, it was also found that *MYB33* and *MYB101* were sensitive to ABA, while miR159 responded to abiotic stressors by suppressing *MYB33* and *MYB101*, affecting seed germination [70]. In our study, miRNAs targeting MYB include novel_1 and novel_11, with novel_1 being highly expressed during dormancy, dormancy release, and germination stages (Tables S8 and S10). It is speculated that the novel_1 target *MYB* may participate in tuber dormancy release by responding to the ABA pathway.

Auxin response factor (ARF) is a type of target in the miRNA160/167 family that is key to converting auxin signals into transcriptional responses. Research has demonstrated that miR160-mediated inhibition of ARF10 is essential for seed germination [27]. During germination in *Arabidopsis* seeds, the mutant of ARF10 displays an upregulation of genes responsive to ABA. Besides, the miR160/ARF module is involved in plant seed germination, which is caused by the mechanism of interaction between auxin of ARF-dependent and ABA [27]. miR167/ARF module can regulate the expression of GRETCHEN HAGEN3 genes, which regulate JA homeostasis and control root initiation [71]. We discovered that the miR160 family has two members (miR160a-3p and miR160a-5p), while the miR167 family has five members (miR167a-3p, miR167b-3p, miR167c-3p, miR167c-5p, and miR167d-3p), all of which target 12 ARF genes (Tables S8 and S10). The stu-miR160a-5p and stu-miR167c-3p expression levels increased as tuber dormancy progressed to sprouting, whereas stu-miR167c-5p expression decreased (Fig. 2D; Table S8), indicating a critical role in the interaction between auxin and ABA pathways during sprouting.

After that, we analyzed the miRNAs targeting *ERF* that were novel_9 and novel_172b-5p (Table S10), of which novel_9 was up-regulated and miR172b-5p was significantly down-regulated from tuber dormancy to germination (Fig. 2D; Table S8). Similarly, previous research described that the AP2/ERF (APETALA2/ET-responsive factor) had been discovered to regulate bud dormancy release [72]. Research has shown that overexpression of *DREB2C* can cause delayed germination of *Arabidopsis* seeds [73]. *SIERF2* transgenic tomato seeds germinated earlier than the WT, increasing the sensitivity of the plants to ET [74]. A study of small RNA and mRNA expression analysis showed that PagmiR172, which targets *AP2*, is differentially expressed in poplar from dormancy to actively growing [75]. It is assumed that these two miRNAs (novel_9 and novel_172b-5p) targeting ERF play a role in tuber dormancy release by responding to the ET pathway. Furthermore, our study demonstrated that the TFs are the predominant targets of most DE-miRNAs, such as miR156/SPL, miR172/AP2, miR159/GAMYB, miR167/ARF, and miR319/TCP (Table S10). In a previous study, it was observed that the DE-miRNAs and their target TFs have been engaged in seed dormancy and sprouting [68, 71]. From the above discussion, it is concluded that the DE-miRNAs and their target TFs are crucial in regulating tuber dormancy release.

### Regulatory role of the stu-miR319c in tuber dormancy release by participating in the jasmonic acid pathway

miR319 is highly conserved and widely presented in plant crops, playing a crucial role in plant growth and development, morphogenesis, and biotic and abiotic stress responses [76]. Moreover, it was the first plant miRNA discovered using a forward genetic mutation screening, and the target genes of miR319 were identified as members of the TCP family, which the existence of miR319/TCP interaction was first revealed in *A. thaliana* [77]. This study verified that stu-miR319c could target *StTCP26* and *StTCP27* and significantly decrease their expression by degradome sequencing, tobacco cotransformation system, and GUS staining three technology (Figs 4D and 5), and our study further showed that the miR319/TCP interaction was highly conserved.

Previous studies demonstrated that the miR319 plays a crucial role in regulating various biological processes, including organ morphology, leaf development, flower development, hormone signaling, and flavonoid synthesis [44]. Similarly, in *Brassica napus*, the miR319 overexpression line exhibits aberrant development of serrated leaves and shoot apical meristem (SAM), leading to stem growth deformation and reduced plant height [78]. It was also reported that interfering with miR319 in wheat (*Triticum aestivum* L.) results in favorable changes in plant structure, including increased plant height and decreased tiller count [79]. The above findings have similar trends to our result, which found that the stu-miR319c transgenic line exhibited changes in morphological traits, such as leaf shape and size, plant height, and root length (Fig. S9B, C, and D; Table S15), suggesting that miR319 regulates these traits and is preserved across species. Additionally, changes in tuber shape, skin smoothness, and bud eye number were stably inherited (Fig. 6A–F), indicating that stu-miR319 plays a crucial role in tuber development and growth, potentially improving the potato crop.

The miR319 has been reported to be involved in important biological processes in *A. thaliana*, rice, tomato, melon (*Cucumis melo* L.), cotton (*Gossypium*), and other species and is essential for the whole developmental stage of plants [44]. Under low-temperature conditions, the reduction of miR319 can promote the cold tolerance of rice and regulate tiller shoot development and grain yield in rice [80]. In cotton, miR319a could impact the elongation of cells and regulate the secondary cell wall formation in fiber cells [81]. In *A. thaliana*, mutant plants of the *HEN1* gene caused a significant reduction in miR319 gene expression, which inhibited shoot regeneration [82]. Our findings validated that the miR319c-OE tubers had sprouted buds when WT tubers were dormancy released, as well as that the miR319c-ST tubers had delayed germination and were still in the dormancy stage (Fig. 6G and H). Altogether, the above statement determined that stu-miR319c has the potential to open up a new avenue for the development of potato varieties with improved sprouting ability.

Multiple studies commenced in recent years have shown the significant function of JA in seed germination and dormancy. Exogenous JA and MeJA have been demonstrated to stimulate the seed dormancy release in multiple species, such as wheat [83], brown mustard [84], *Acer tataricum* [85], apple (*Malus pumila* Mill.) [86], and Douglas fir [*Pseudotsuga menziesii* (Mirb.) Franco] [87], while exogenous JA prevents nondormant seeds from germinating, including *Arabidopsis* [88], rapeseed (*B. napus,*), and flax (*Linum usitatissimum*) [89]. In our investigation, exogenous MeJA was utilized to treat WT tubers, and it was discovered that low concentrations of MeJA significantly broke dormancy and enhanced the sprouting of potato tubers (Fig. 7A–C). A previous report exhibited that a drop in JA content was seen simultaneously with the dormancy release of potato tuber, and a fast rise in JA content was observed concurrently with the beginning of sprouting [90], which showed more consistency with our investigations.

**Figure 8:**
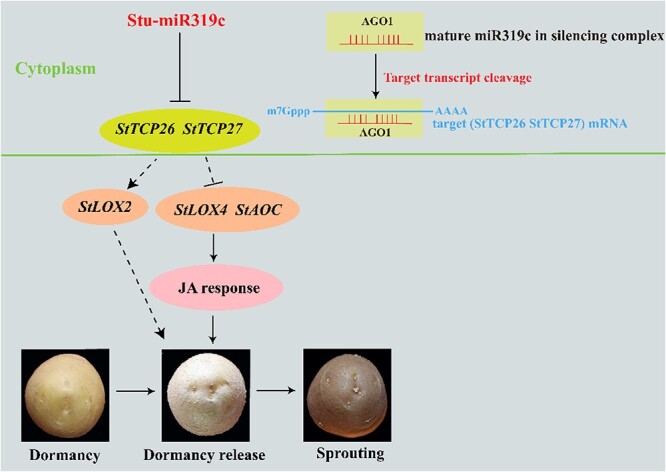
Potential model for miR319 regulating tuber dormancy release in potato. The miR319c participates in the JA pathway to regulate tuber dormancy and sprouting. Arrows show simultaneous effects in the pathway, while nail shape represents repression. The solid lines indicate results obtained from this study or previous studies, and the dashed lines indicate hypothetical pathways affecting tuber dormancy release based on the change of expression. Mature miRNA-cleaving target genes occur in the cytoplasm [94]. microR319c mature body forms a complex with the AGO proteins, binds to the target genes (*StTCP26*, *StTCP27*), and cleaves the target genes at the most intermediate binding site of the microRNA. When the mRNA is degraded into fragments, the function of its encoded proteins also disappears [95]. StLOX4 (13-type lipoxygenase) and StAOC promote the synthesis of JA [96]. Our study and Park [90] reported that JA promotes tuber dormancy release. The target (StTCP26, StTCP27) of miR319c regulate genes (*LOX2*, *LOX4*, and *AOC*) associated with the JA synthesis pathway, which is inferred by measuring the expression of miR319c transgenic plants.

13-LOX catalyzes the formation of 13-HPOT (13-hydroperoxy-linoleic acid) from the JA synthesis precursor, and AOC (allene oxide cyclase) catalyzes the formation of OPDA from 13-HPOT, both of which are essential enzymes in the JA synthesis pathway [48]. Previous research has shown that overexpression of *OsLOX2* can promote rice seed germination under normal conditions [91]. AOC promoter transcription is enhanced by *OsbZIP72,* resulting in an increased content of endogenous JA and inhibiting seed germination [92]. Previous studies reported that the miR319/TCP module is engaged in various developmental processes, primarily through impacted JA synthetic genes and the endogenous JA level [47]. Our study on transgenic tubers revealed significant changes in JA content and expression levels of JA synthesis genes (*StAOC*, *StLOX2*, and *StLOX4*) (Fig. 7D and E), suggesting that the miR319c may participate in the JA pathway to regulate tuber dormancy release**.** The *LOX2* and *LOX4* genes measured in this study belong to the same LOX enzyme family. However, they exhibited distinct patterns of expression in the stu-miR319c transgenic tubers. This is due to the categorization of LOX into 9-type and 13-type, with 13-type lipoxygenase engaged in JA synthesis [93], which supports our results as the *LOX4* gene belongs to the 13-type and increases JA synthesis in our study, while the *LOX2* gene, which belongs to the 9-type, is not engaged in JA synthesis and is solely thought to be involved in regulating tuber dormancy release (Fig. 8). The study reveals stu-miR319c as a unique epigenetic regulator involved in potato tuber dormancy release and uncovers new concepts on the regulation mechanism governing potato tuber dormancy release. In addition, it is essential to acknowledge that the regulation of tuber dormancy release and sprouting by stu-miR319c is intricate. We cannot exclude the possibility that miR319c may regulate other target genes, and the function of the target genes *TCP* (*StTCP26* and *StTCP27*) in potato tuber dormancy release has not yet been validated, with TCP being reported to be involved in dormancy release in other crops [97] and only one paper in potato [98]. To address this significant issue, we plan to conduct a separate and dedicated study in the future to investigate the function of the target genes (*StTCP26* and *TCP27*) of miR319c in regulating tuber dormancy release and to explore the mechanism of miR319c regulation during potato tuber dormancy release.

## Conclusion

In conclusion, we identified 204 known and 192 novel miRNAs from six libraries of potato tubers. A total of 113 DE-miRNAs were screened during the dormancy to sprouting period, and a total of 56 DE-miRNAs were screened and regulated by temperature during tuber dormancy release. The result of degradome sequencing and tobacco cotransformation system assay indicated that the *StTCP26* (Soltu.DM.08G007340) and the *StTCP27* (Soltu.DM.08G007360) are the downstream target genes of stu-miR319c. Additionally, compared with WT plants, stu-miR319c transgenic plants had significant differences in leaves, plant height, and root length, and the transgenic tubers also had changes in the smoothness of the potato skin, tuber shape, and the number of bud eyes. This study also found that stu-miR319c can act as a positive regulator of tuber dormancy release. Treatment of WT tubers with exogenous MeJA (0, 0.5, 1, 2, and 4 mg/L) demonstrated that 1 mg/L was the most effective concentration to promote the dormancy release of tubers and significantly improved the sprouting rate of tubers. The result of JA content and relative expression of JA biosynthesis–related key genes in miR319c transgenic tubers suggested that the miR319c may participate in the JA pathway to regulate tuber dormancy release. Overall, our study lays the foundation for elucidating how miRNA regulates tuber dormancy release and provides new insight into the functional analysis of the miR319c that regulates the dormancy-sprouting transition in potato tuber.

## Materials and methods

### Plant materials

The potato cultivar ‘Atlantic’ used in this study was grown in Xindian Town, Lintao County, Dingxi City, Gansu Province of China. The potatoes were planted in May 2020, and tubers were harvested in September 2020. The tubers with the same size, uniform shape, and health were selected and stored in darkness at 12°C for a while until they were fully mature and promoted wound healing. Then, they were placed in light-proof incubators at 4 and 22°C, respectively, for dormancy release and sprouting. Fully mature tubers (0d) were defined as DTs. The DRTs were defined according to the highest expression period of dUTPase meristem genes during the dormancy release process of tubers [99], and those with bud lengths of 2–3 mm long were defined as sprouting tubers (ST). DT, DRT, and ST samples were collected from tubers that were stored at 4 and 22°C, respectively, by utilizing the techniques outlined by Liu *et al* [19]. The primers used in this study are listed in Table S16.

### Small RNA libraries and degradome library construction and sequencing

Total RNA was extracted from DT to ST potato tuber eyes stored at 4 and 22°C using the TRNzol Universal Reagent (TIANGEN Biotechnology, China), and the purity, concentration, and integrity of the RNA were checked by NanoPhotometer® spectrophotometer (IMPLEN, USA), Qubit® 2.0 Fluorometer (Life Technologies, USA), and Agilent Bioanalyzer 2100 system (Agilent Technologies, USA). After the samples were qualified, part of the samples was sent to Novogene Biotechnology Co., Ltd. (Beijing, China) to construct an sRNA library, and single-end sequencing was performed using the Illumina SE50 sequencing system (Novogene Bio, China). Another part of the samples was sent to Lianchuan Biotechnology Co., Ltd. (Hangzhou, China) to construct a degradome library, and single-end sequencing was performed using Illumina Hiseq2000/2500 (LC Bio, China).

### Analysis of sequencing data

To analyze the miRNA sequencing data, clean reads were first obtained by processing raw reads. Length-screened sRNA (18–30 nt) was mapped to the potato genome using Bowtie-0.12.9 [100] to analyze the distribution of sRNAs over reference sequences. These reads were mapped to the reference sequences and compared to the mature potato miRNA sequences (miRBase 21, http://www.mirbase.org/) to discover known conserved miRNAs. The sRNAs obtained from sequencing were annotated using the Rfam database (http://rfam.xfam.org/) to identify possible rRNAs, tRNAs, snRNAs, and snoRNAs among them. The reads that were mapped to the reference genome and that were not matched to the RNA (rRNAs, tRNAs, snRNAs, snoRNAs) were used for further analysis. According to the iconic hairpin structure of miRNA precursors, the miRNA prediction software miREvo_v1.1 [101] and mirdeep2_0_0_5 [102] were used to analyze novel miRNAs. Based on the read count data obtained by the analysis of miRNA expression levels. DESeq2 [103], with a negative binomial distribution, was employed to determine the differential expression of miRNA. The screening criteria for differential genes are critical. To control the false-positive rate, the screening conditions with padj < 0.05 and |log2(foldchange)| > 1 were set to obtain differential expression miRNAs.

For degradome sequencing data, the raw data were trimmed by FastQC to obtain clean reads. Then, the reads were mapped to the potato complementary DNA (cDNA) database, and the mRNA sequences of target genes matched with potato miRNA sequences were predicted by the splicing site prediction software (GSTAr). The spliced miRNA targets were identified and classified according to the CleaveLand 4.0 version program [104].

### Stu-miR319c characteristics and its target genes

The recombinant vectors containing the GUS (β-glucuronidase) reporter gene received injections into the leaves of *N. benthamiana* via the *Agrobacterium*-mediated transfection system (GV3101). Positive controls included pBI121-GUS and a mixture of pBI121-GUS and pBI121-miR319c, while the negative control was pBI121-miR319c. pBI121-GUS, pBI121-*StTCP26-*GUS, pBI121-*StTCP27-*GUS, and pBI121-miR319c were cultured to OD_600_ = 0.6–0.8 before injection. The injected plants were then grown under dark conditions at room temperature for 2–3 days. GUS histochemical staining was evaluated in three separate biological replications.

### Cloning and subcellular localization of StTCP26 and StTCP27

For subcellular localization, we amplified the open reading frames of StTCP26 and StTCP27 without terminators, and constructed the positive vectors (StTCP26-EGFP and StTCP27-EGFP), using the homologous recombination method by ligating the terminator-denuded ORFs to the pCAMBIA1300 vector containing EGFP protein. StTCP26-EGFP, StTCP27-EGFP, and EGFP were cultured to OD_600_ = 0.6–0.8 before injection. The injected plants were then grown under dark conditions at room temperature for 2–3 days. EGFP fluorescence had been viewed with a laser-scanning confocal microscope LSCM 800 (CARI ZEISS, Germany) [105].

### Vector construction and generation of transgenic potato plant lines

To investigate the function of differentially expressed miRNAs identified during potato tuber dormancy release, artificial miRNA and short-tandem target mimic (STTM) technologies were used to overexpress or silence the expression of miR319c during potato tuber dormancy release. For overexpression of miR319c, the hairpin sequence of the stu-miR319c sequence (pre-miR319c) was amplified using specific primers (Table S16), and the fragment was inserted into the pCAMBIA1300 vector under the control of 35S promoter. For ST of miR319c, STTM vector was constructed by the following procedure described by Teotia *et al* [106]. Using the binding site of stu-miR319c on the target gene as the target repetitive sequence, a 48 nt sequence is connected in the middle, with two miR319c binding sites at each end, to synthesize a miR319c-STTM fragment. The fragment was inserted into the pCAMBIA1300 vector under the control of 35S promoter.

Potato transformation was performed using the *Agrobacterium*-mediated technique for stem segment and potato slice transformation [107]. Potato ‘Atlantic’ tissue culture plants that had grown for ~4 weeks were selected, and sterile scissors were used to cut a 1 cm long stem segment without axillary buds from the tissue culture seedlings. The stem segments were placed in MS solid medium (2 mg/L IAA + 1 mg/L 6-BA, 3% sugar) and precultured in a constant temperature incubator for 2 days. The potato ‘Atlantic’ micro-tubers were peeled and cut into potato slices with a 1–2 mm thickness. Furthermore, the potato chips and the stem segments precultured for 2 days were infected with *Agrobacterium tumefaciens* containing pCAMBIA1300-miR319c-OE and pCAMBIA1300-miR319c-STTM for 10 min, respectively. The bacterial solution on the surface of stem segments and potato slices was blotted dry with sterile filter paper and placed in MS solid medium (2 mg/L zeatin (ZT) + 1 mg/L IAA + 0.5 mg/L 6-BA +0.2 mg/L GA3 + 50 mg/L Kan, 3% sugar) and then cocultured for 2 days in a dark incubator at 28°C. The cocultured materials were transferred to a differentiation medium (MS + 2 mg/L ZT + 1 mg/L IAA + 0.5 mg/L 6-BA +0.2 mg/L GA3 + 100 mg/L Cef，3% sugar) and differentiation culture in a light chamber (16 h light/8 h dark with a light intensity of 20 000 lx) at 25°C. When the new buds were generated from the callus of the stem segments and potato slices, they were transferred into rooting MS medium (100 mg/L Hyg + 50 mg/L Cef) for screening hygromycin-resistant transformed plants. After three times of subculture screening, the candidate transgenic plants were preliminarily obtained. Positive transformations plants for miR319c overexpression and interference expression transgenic lines were detected by PCR using hygromycin-specific primers, with DNA from WT potato plants as a negative control and pCAMBIA1300-miR319c-OE and pCAMBIA1300-miR319c-STTM transformed plants as experimental groups. The relative expression levels of the stu-miR319c gene in different transgenic lines were detected by qRT-PCR. The OE-S4, OE-S5, and OE-S6 lines with high expression levels in miR319c-OE transgenic plants and the ST-T10, ST-T11, and ST-T19 lines with low expression levels in miR319c-STTM transgenic plants were selected for the subsequent functional study of stu-miR319c gene. A spiral micrometer was used to measure leaf length, leaf width, plant height, root length, tuber length, and tuber width. An electronic balance was used to measure plant fresh weight, fresh root weight, and tuber weight.

### Dormancy period test and sprouting analysis

To comprehend the function of stu-miR319c in potato dormancy release and sprouting, miR319c transgenic and nontransgenic potato plants were grown in greenhouses with coconut bran substrate in Xindian Town, Lintao County, Dingxi City, Gansu Province of China. Before planting, the tissue culture plantlets growing for ~3 weeks need to be refined. Firstly, the plantlets should be placed with natural light (avoiding the sun from 1 to 3 p.m.) without opening the tissue culture bottle cover to grow for 4 days, and then, the tissue culture bottle should be opened and grown for 3 days in the same environment. At this time, the plant has adapted to natural light and is planted in a moist coconut bran substrate in the greenhouse for 2 years. In the first year, it was planted on 28 April 2022 and harvested on 2 September 2022. In the second year, it was planted on 15 May 2023 and harvested on 18 September 2023. The tubers were harvested ~120 days during the growth period. The tubers go through wound healing for 2 weeks at (22 ± 1)°C in the dark after harvest and, subsequently, 30 tubers for each transgenic line were selected for dormancy testing. The tubers used for the dormancy test were placed in a box (5 L) and stored in the dark at room temperature (22 ± 1)°C. A tuber is considered sprouted when it has a sprout that is at least 2 mm long. The sprouting time was recorded at 4-day intervals until all tubers had sprouted. The samples from DT, DRT, and ST periods were collected for qRT-PCR and hormone content determination experiments.

### Effects of jasmonic acid treatment on dormancy release and sprouting of tubers

To study the effect of JA on dormancy release and sprouting of tubers, ~200 dormant tubers of uniform size were selected from the nontransgenic pre-elite seeds of the ‘Atlantic’ variety. The dormant tubers were immersed in different gradients of MeJA (Solarbio Biotechnology, China) at 0 (control), 0.5, 1, 2, and 4 mg/L for 30 min and then dried and placed in the dark at room temperature (22 ± 1)°C. The sprouting time was recorded until all tubers had sprouted. After 8 and 16 weeks of treatment, the sprouting was observed, and the bud length was counted. Twenty tubers were used per treatment, and the experiment was repeated twice.

### Determination of endogenous jasmonic acid content

To verify the regulation of stu-miR319c for tuber dormancy release and sprouting by the JA pathway, JA was extracted from bud-eye tissues of miR319c transgenic and nontransgenic tubers during the dormancy release period. The JA content was measured as described in previous literature [108] and detected by an Agilent 1290-6460 LC-MS/MS instrument (Agilent Technologies, USA). Each sample was characterized based on three replicates.

### Quantitative real-time PCR assays

A qRT-PCR test was conducted to confirm the miRNA expression. Total RNA was extracted using the Trizol method, and cDNA was synthesized by using the miRNA first-strand cDNA synthesis kit (Accurate Biotechnology, China). St18S RNA was employed as an internal control. To quantify miRNA target genes, total RNAs were reversely transcribed by the Evo M-MLV RT Kit (Accurate Biotechnology, China), and the *Stef1α* gene was used as a reference gene for normalization. Quantitative PCR was performed using the SYBR^®^ Green Premix Pro Taq HS qPCR Kit (Accurate Biotechnology, China) in a Light Cycler® 96 system (Roche Applied Science, Germany). The relative expression levels of miRNAs and genes were both calculated using the 2^−ΔΔCt^ method [109].

### Statistical analysis

All data were analyzed using SPSS 22.0 (IBM, USA) for statistical testing. Samples were analyzed in biological triplicate, and the data represented mean ± standard deviation. Statistical significance was evaluated using one-way analysis of variance followed by Tukey’s test or Dunnett test (*P <0.05, **P <0.01). Bars with distinct letters indicate statistically significant differences in means (*P* <0.05). All figures were made using Origin 2021 (OriginLab Technologies, USA) and Adobe Illustrator 2020 (Adobe Technologies, USA) software.

## Supplementary Material

Web_Material_uhae303

## Data Availability

The raw sRNA sequencing and degradome sequencing data reported in the present study have been deposited in the National Center for Biotechnology Information (NCBI) database under project numbers PRJNA1160163 and PRJNA1160198, respectively.
