# Supplementary material for: Genome-wide analysis of non-coding RNA reveals the role of a novel miR319c for tuber dormancy release process in potato
Source: Hortic Res. 2024 Oct 30;12(2):uhae303. doi: 10.1093/hr/uhae303 (PMC11822407; doi:10.1093/hr/uhae303)
Supplement: Web_Material_uhae303 [file web_material_uhae303.zip › Supplementary Figures-R2.docx]

**Supplementary**

**Figure S1.** The unique length distribution of sRNA.

**Figure S2.** Identification and length distribution of known and novel miRNAs in potato.

**Figure S3.** Base preference analysis of miRNAs in potato tubers.

**Figure S4.** Validation of the sRNA-Seq results using qRT-PCR.

**Figure S5.** Analysis of KEGG pathways and GO terms of the target genes of tuber dormancy release related stu-miRNAs.

**Figure S6.** Secondary structure for stu-miRn220.

**Figure S7.** Subcellular localization of StTCP and expression pattern of stu‑miR319c/StTCP in various tissues.

**Figure S8.** Construction of Stu-miR319c overexpression and interference expression vectors and *Agrobacterium*-mediated genetic transformation.

**Figure S9.** Phenotypes of stu-miR319c transgenic plants.

**Table S1.** Summary of the data quality of miRNA sequencing.

**Table S2.** Number and percentage of clean reads mapped to the potato genome.

**Table S3.** Number of sRNA unique reads annotations.

**Table S4.** Types of sRNA unique reads annotations.

**Table S5.** Statistics of known and novel miRNA comparisons.

**Table S6.** Information on tuber dormancy release related stu-miRNA.

**Table S7.** Conserved miRNA families in potato.

**Table S8.** Differentially expressed miRNAs in tuber dormancy release.

**Table S9.** Target genes predicted of miRNAs by psRobot.

**Table S10.** Target genes of degradome sequencing.

**Table S11.** GO enrichment analysis of miRNA target genes obtained by degradome sequencing.

**Table S12.** KEGG enrichment analysis of miRNA target genes obtained by degradome sequencing.

**Table S13.** Phytohormone pathway-related miRNAs involved in tuber dormancy release and sprouting.

**Table S14.** Related information of stu-miRn220 target gene.

**Table S15.** Phenotypic analysis of the stu-miR319c transgenic plants.

**Table S16.** The primers used in the present study.

**Supplementary Figure**


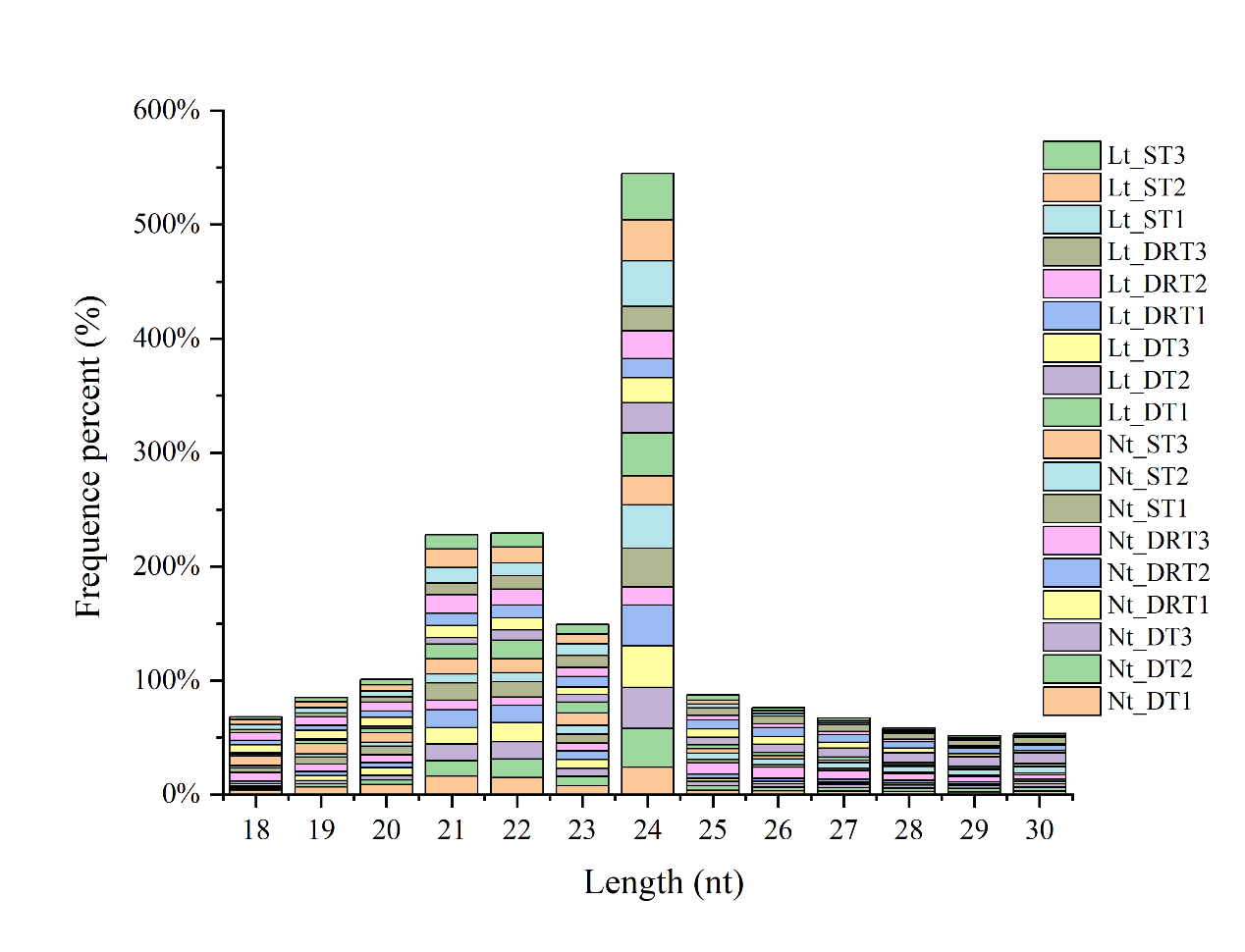


**Figure S1** **The unique length distribution of sRNA.** From 18 nt to 30 nt length sRNA were counted.


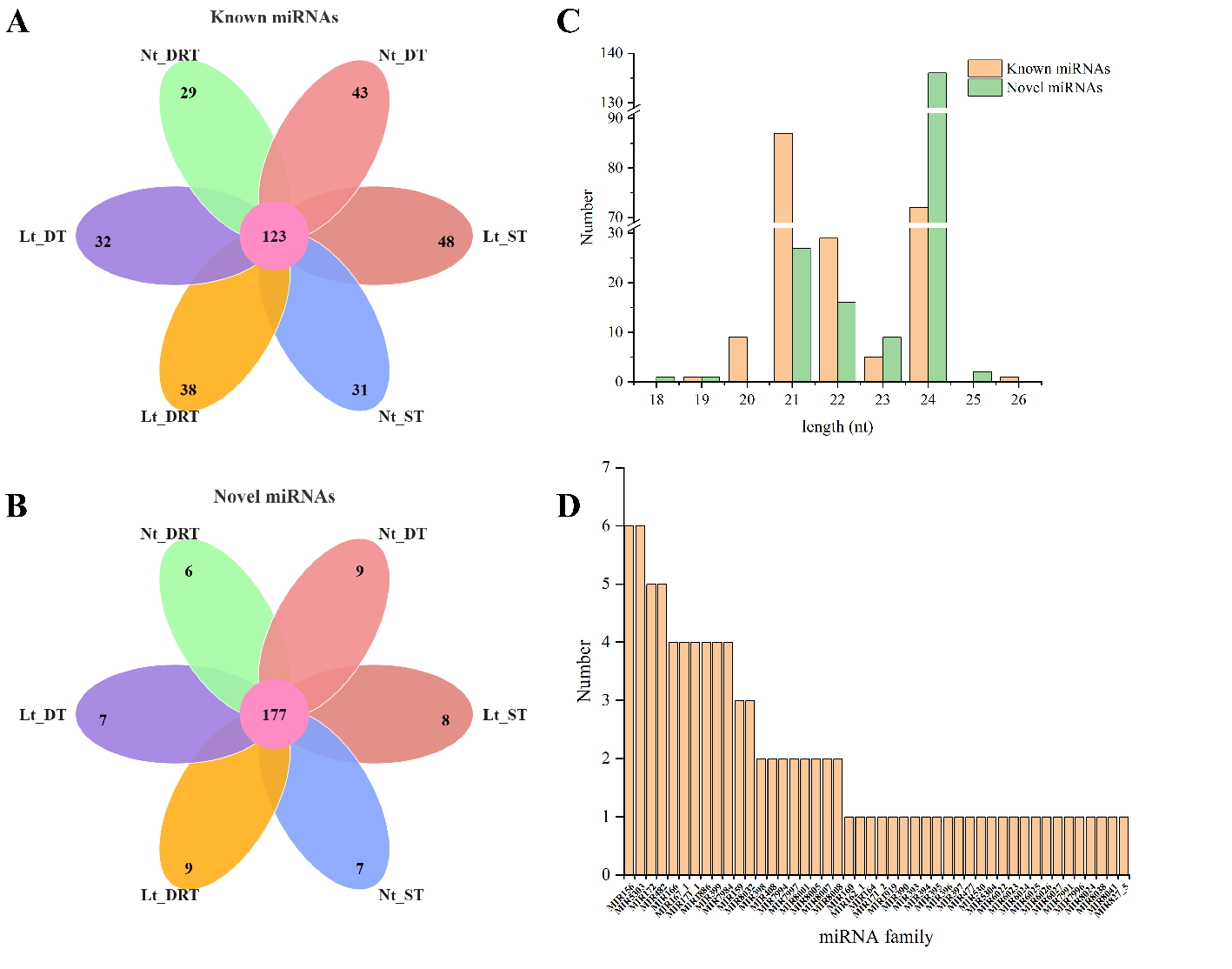


**Figure S2** **Identification and length distribution of known and novel miRNAs in potato.** (A) Flower plot of known miRNAs identification. (B) Flower plot of novel miRNAs identification. (C) Length distribution of known and novel miRNAs. (D) Conserved miRNA families in potato.


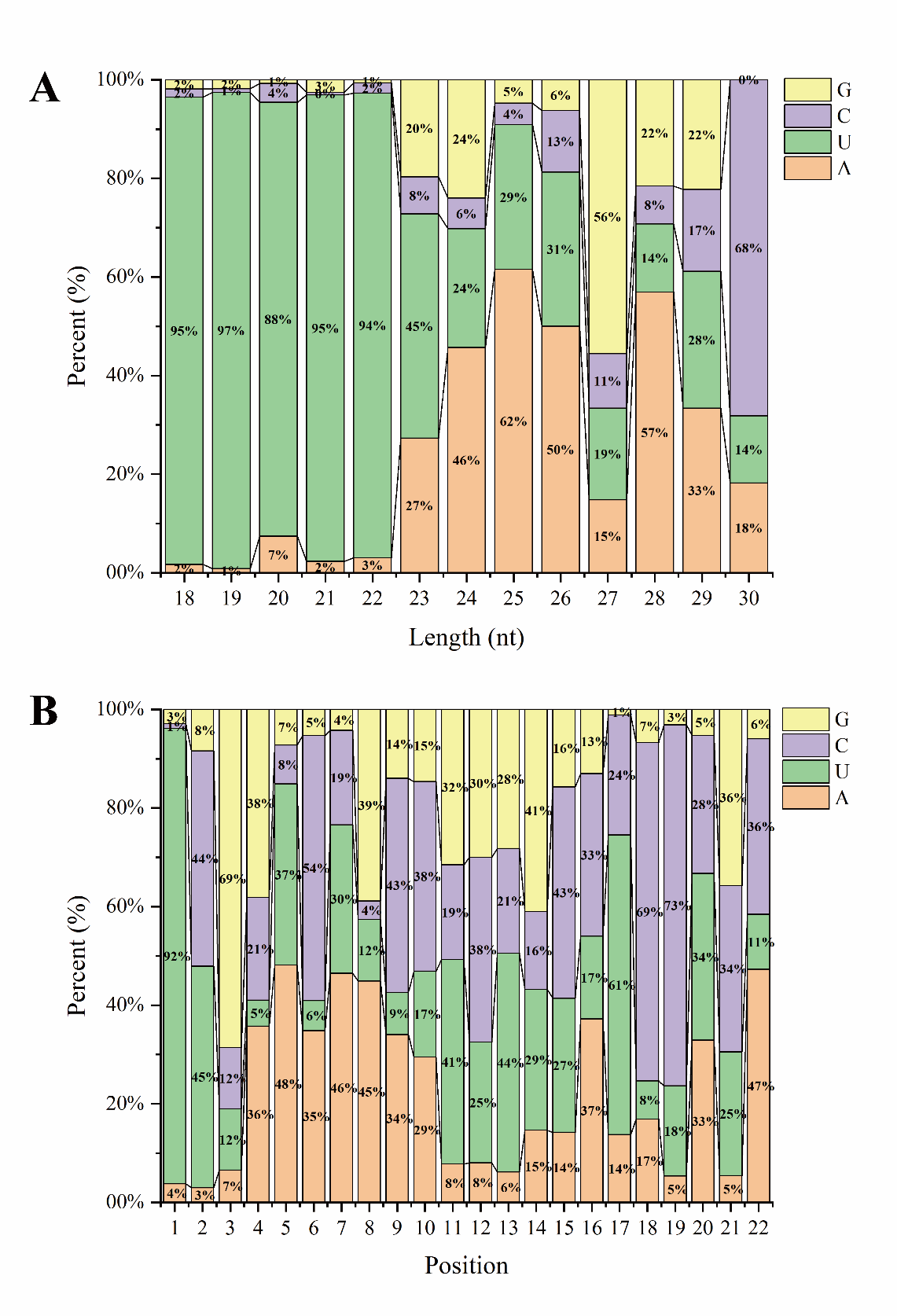


**Figure S3** **Base preference analysis of miRNAs in potato tubers.** (A) the first base preference of miRNAs with different lengths. (B) base preference of miRNAs per site.


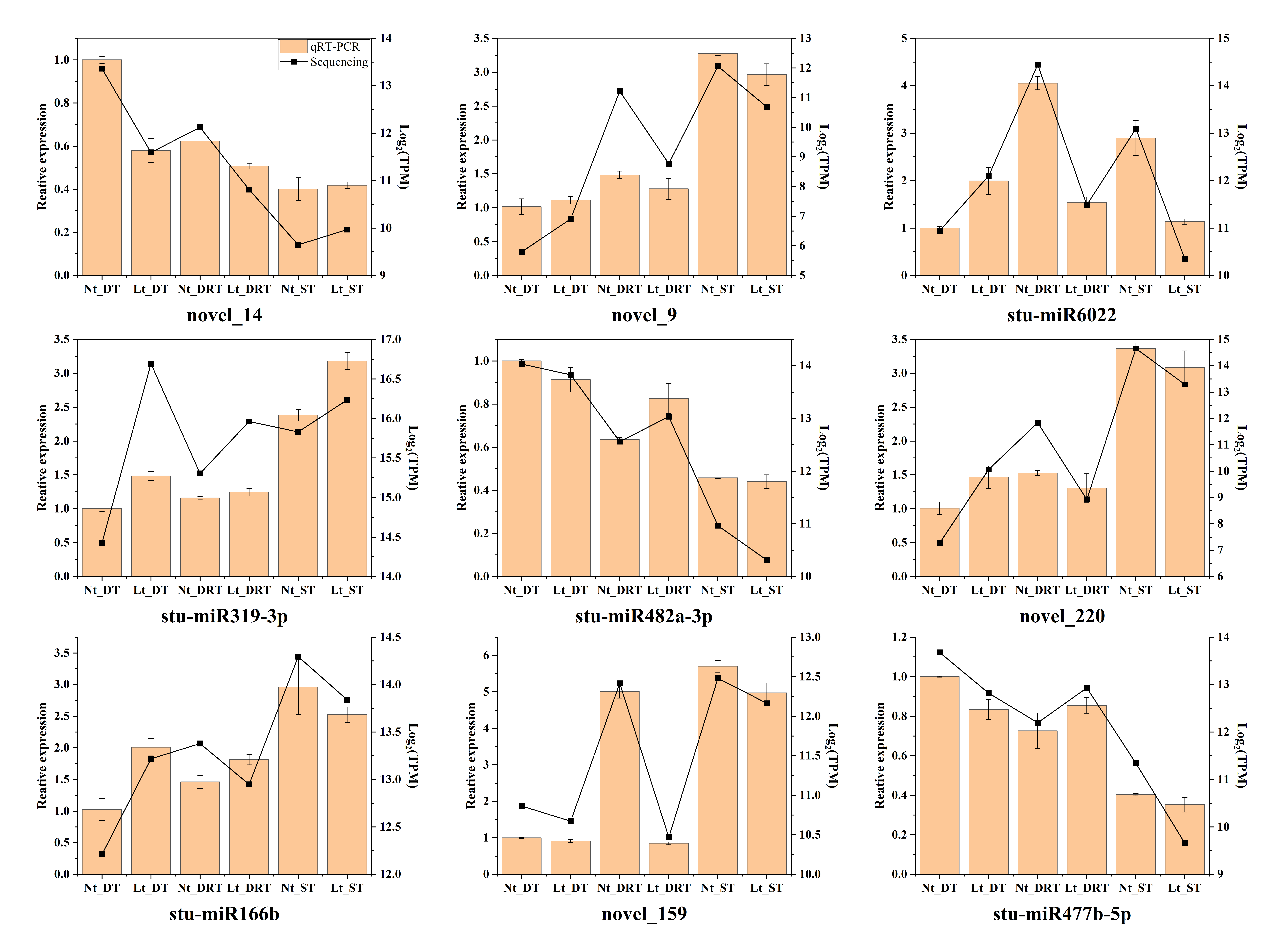


**Figure S4 Validation of the sRNA-Seq results using qRT-PCR.** Left and right Y axis represent the expression level of miRNAs from qRT-PCR and sRNA sequencing data, respectively. Data represent the mean ± SD (n = 3) of three biological replicates.


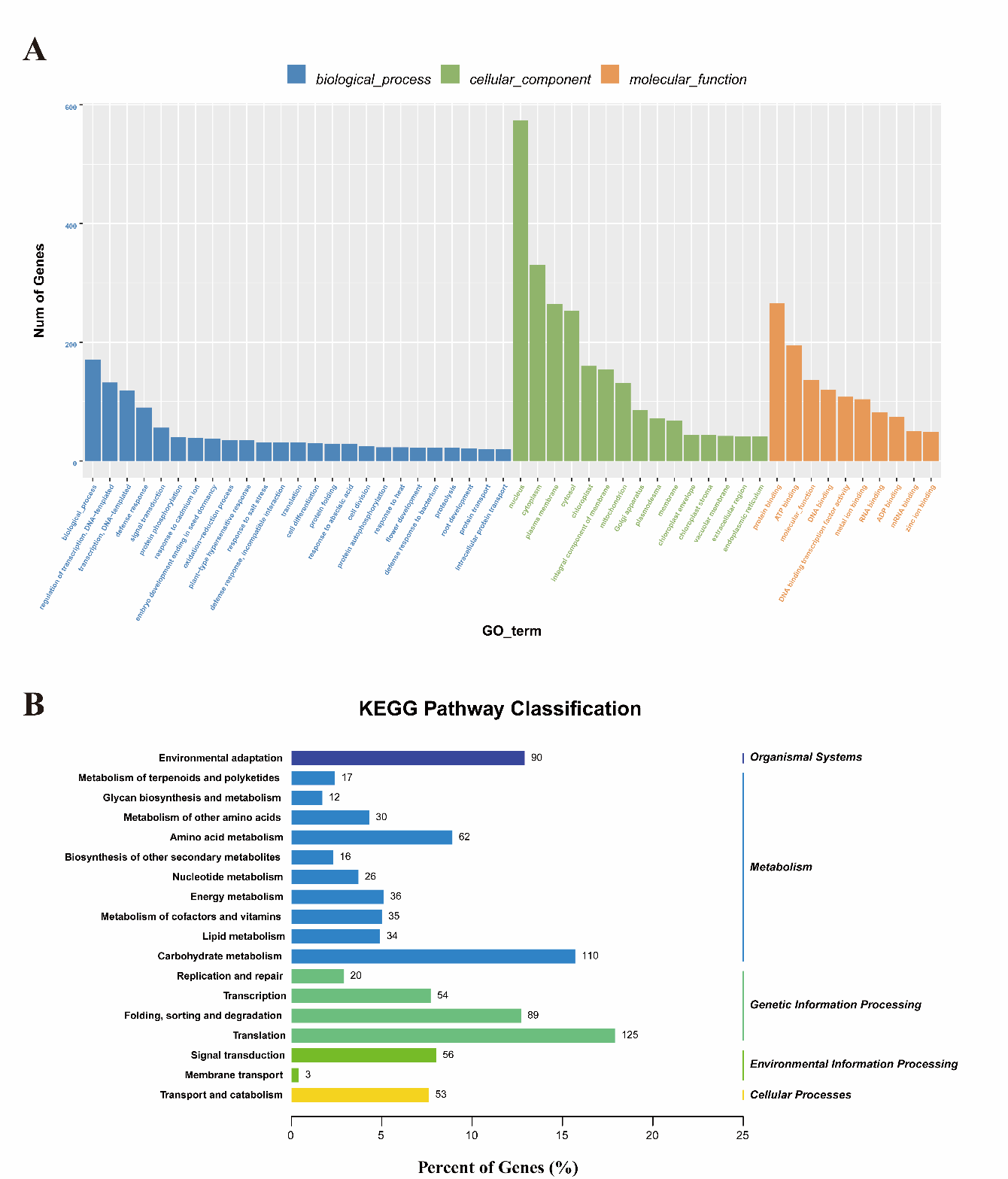


**Figure S5 Analysis of KEGG pathways and GO terms of the target genes of tuber dormancy release related stu-miRNAs.** (A) The proportion of major GO pathways of target genes obtained by degradome sequencing. (B) The proportion of main KEGG pathways of target genes obtained by degradome sequencing.


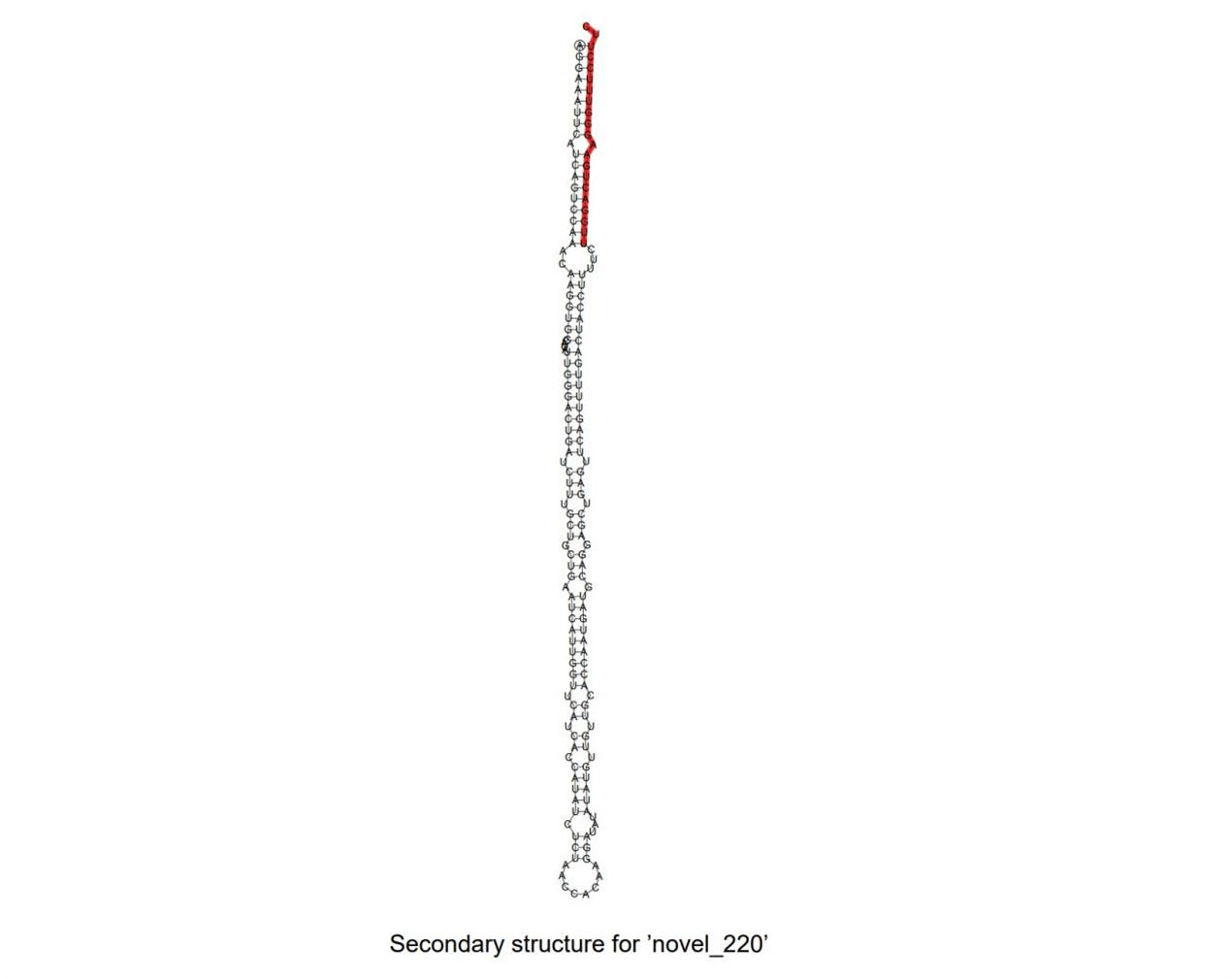


**Figure S6 Secondary structure for stu-miRn220.**


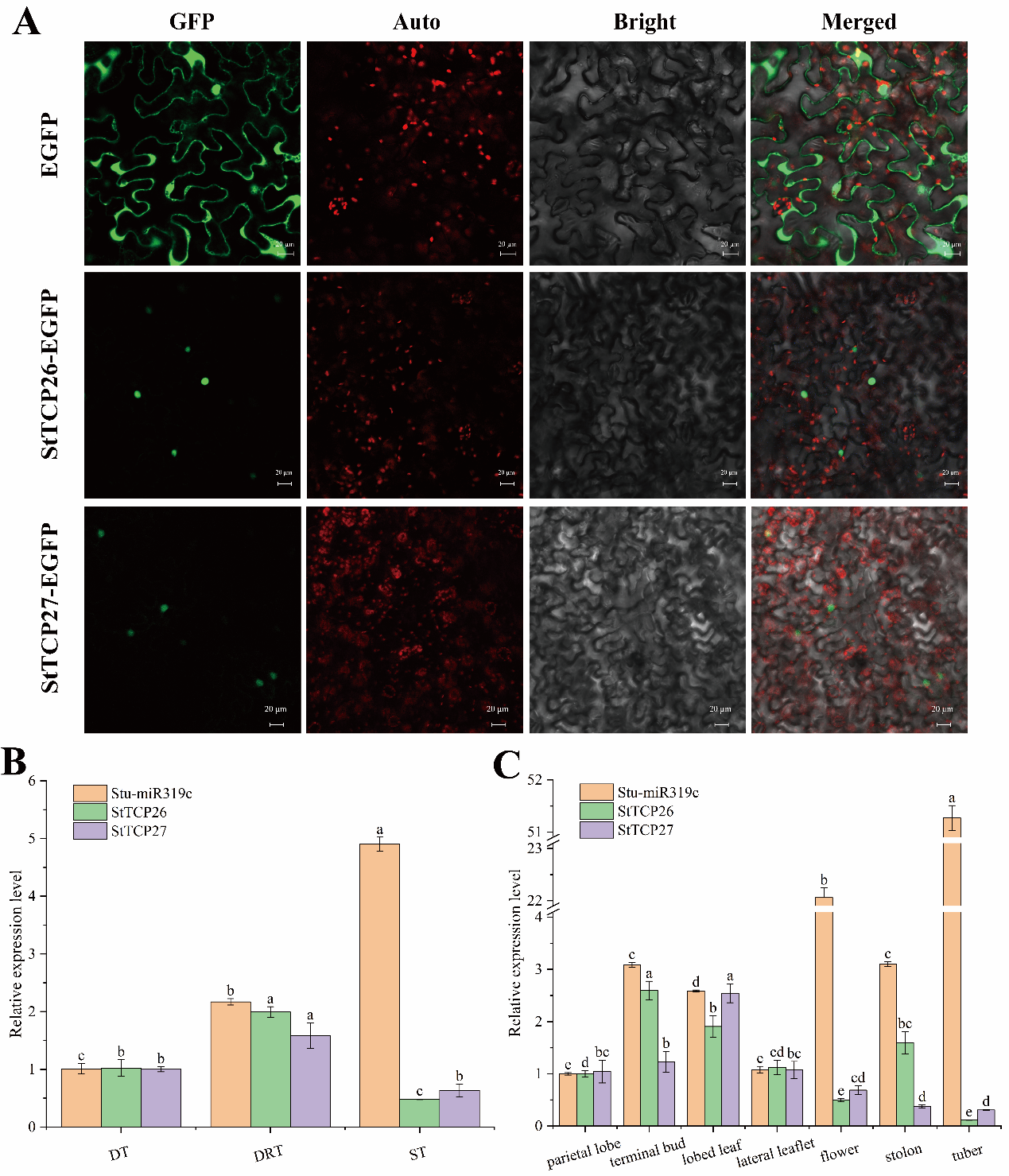


**Figure S7** **Subcellular localization of StTCP and expression pattern of stu‑miR319c/StTCP in various tissues.** (A) Subcellular localization of StTCP26 and StTCP27. The EGFP, StTCP26-EGFP, and StTCP27-EGFP constructs were each transiently expressed in tobacco leaves. The scale bale represents 20μm. The images contain green fluorescent protein field, chlorophyll auto-fluorescent signal, bright field, and merged microscope images. (B) Expression patterns of stu‑miR319c/*StTCP* in the three stages during the dormancy release process of tubers. (C) Tissue-specifc expression patterns of stu‑miR319c/*StTCP* in potato plant. The data represented mean ± standard deviation. Lower case letters indicate the significant difference (at *P* < 0.05) based on Tukey’s test.


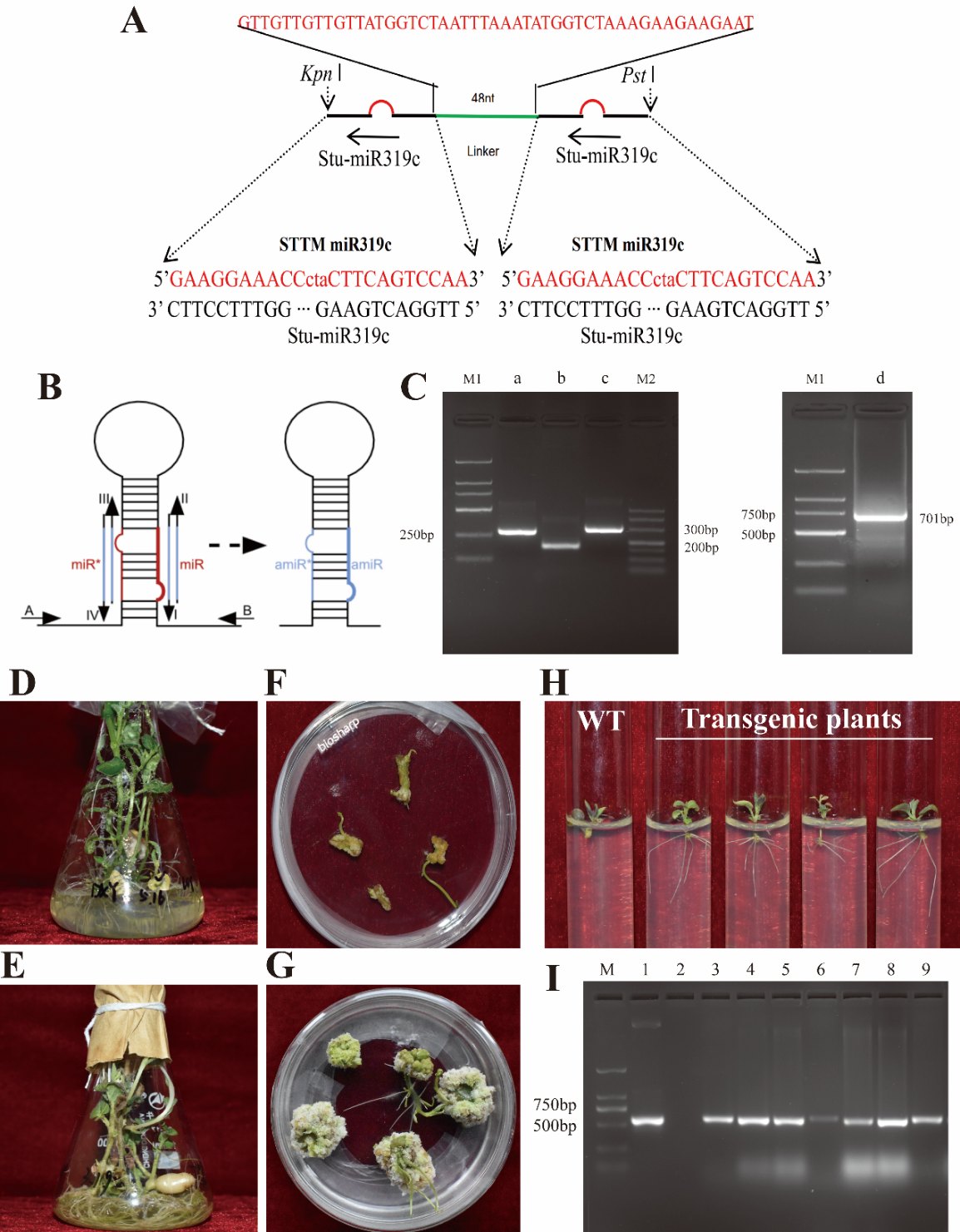


**Figure S8 Construction of Stu-miR319c overexpression and interference expression vectors and *Agrobacterium*-mediated genetic transformation.** (A) Stu-miR156 STTM vector map. (B) Schematic diagram of artificial miRNA precursor sequence amplification. (C) PCR amplification of the precursor fragment. (D-E) tissue culture Seedlings and mini tubers of the *Atlantic* variety. (F-G) Stem segments and tuber callus differentiated into buds. (H) Screening of transformed plants by rooting. (I) Amplified PCR product (566 bp) of *Hyg* gene in transgenic plant. M: DL 2000 marker; 1: positive control (pCAMBIA1300 plasmid); 2: negative control (WT); 3-9: stu-miR319c transgene lines.


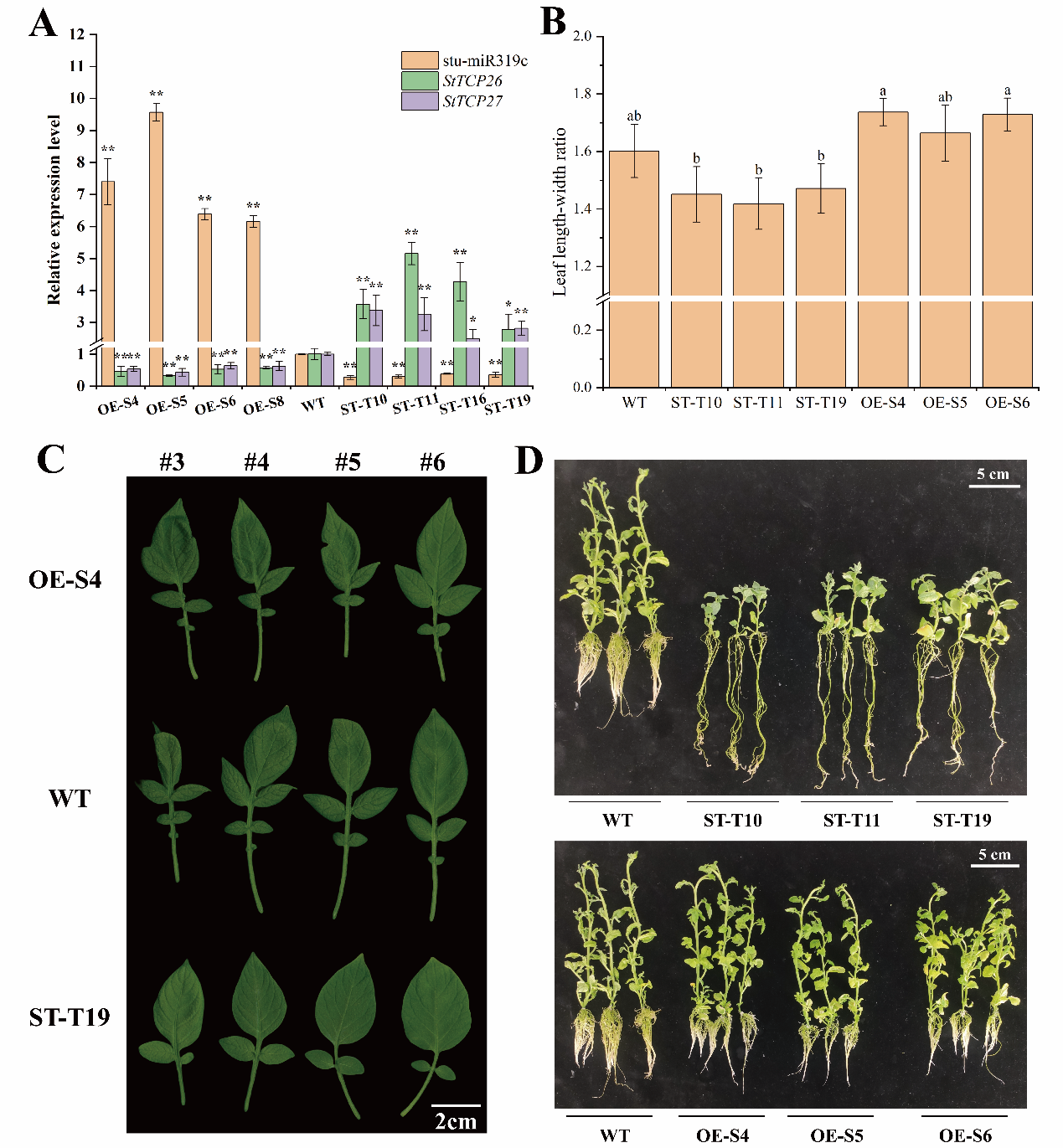


**Figure S9 Phenotypes of stu-miR319c transgenic plants.** (A) Relative expression of stu-miR319c and target genes in transgenic plants. (B) Leaf length-width ratio. (C) Leaf shape. (D) Morphological characteristics of transgenic plants. All data represented the mean ± standard deviation. P-value（**P* < 0.05; ***P* < 0.01）were calculated by Dunnett test. Lower case letters indicate the significant difference (at *P* < 0.05) based on Tukey’s test.
